# Magnetic Biotransport: Analysis and Applications

**DOI:** 10.3390/ma3042412

**Published:** 2010-03-30

**Authors:** Edward P. Furlani

**Affiliations:** Institute for Lasers, Photonics and Biophotonics, 432 Natural Science Complex, University at Buffalo (SUNY), Buffalo, NY 14260, USA; E-Mail: efurlani@buffalo.edu; Tel.: +1-716-645-4150; Fax: +1-716-645-6945

**Keywords:** magnetic biotransport, magnetophoresis, magnetic nanoparticle transport, magnetic drug targeting, magnetofection, bioseparation

## Abstract

Magnetic particles are finding increasing use in bioapplications, especially as carrier particles to transport biomaterials such as proteins, enzymes, nucleic acids and whole cells *etc*. Magnetic particles can be prepared with biofunctional coatings to target and label a specific biomaterial, and they enable controlled manipulation of a labeled biomaterial using an external magnetic field. In this review, we discuss the use of magnetic nanoparticles as transport agents in various bioapplications. We provide an overview of the properties of magnetic nanoparticles and their functionalization for bioapplications. We discuss the basic physics and equations governing the transport of magnetic particles at the micro- and nanoscale. We present two different transport models: a classical Newtonian model for predicting the motion of individual particles, and a drift-diffusion model for predicting the behavior of a concentration of nanoparticles that takes into account Brownian motion. We review specific magnetic biotransport applications including bioseparation, drug delivery and magnetofection. We demonstrate the transport models *via* application to these processes.

## 1. Introduction

**Figure 1:**
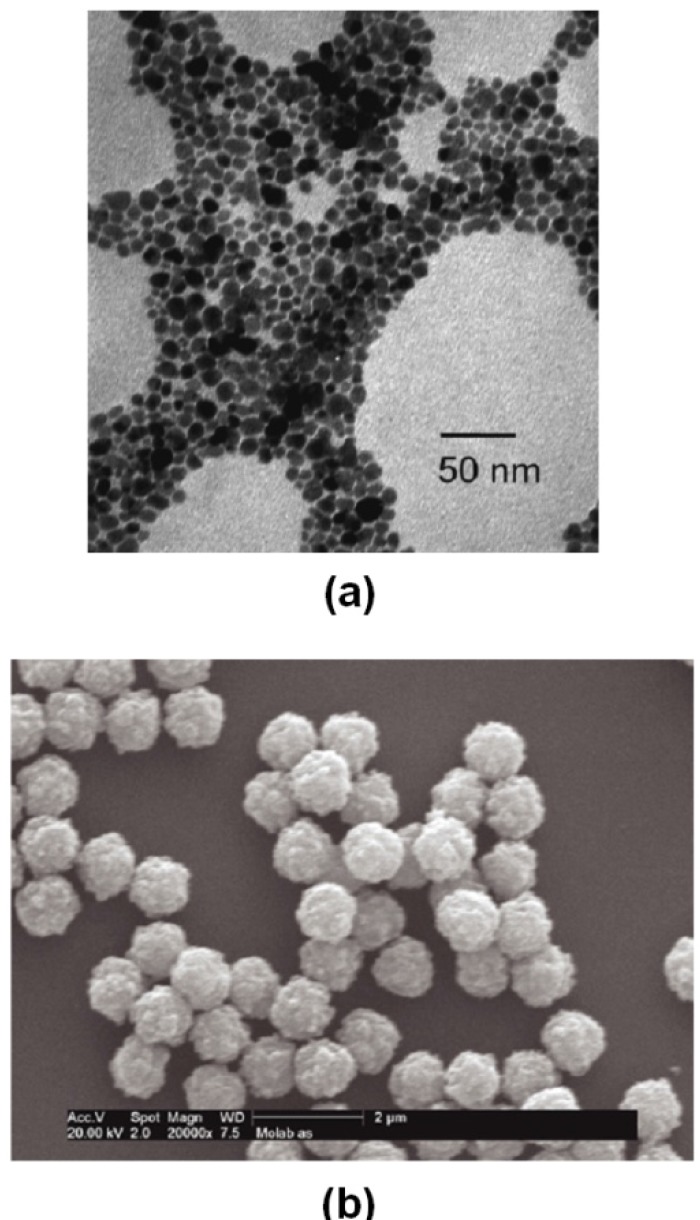
Magnetic particles: (a) TEM of Fe_3_O_4_ nanoparticles without size selection. (b) polymeric microparticles with embedded magnetic nanoparticles (Dynabeads from Dynal Biotech).

**Figure 2:**
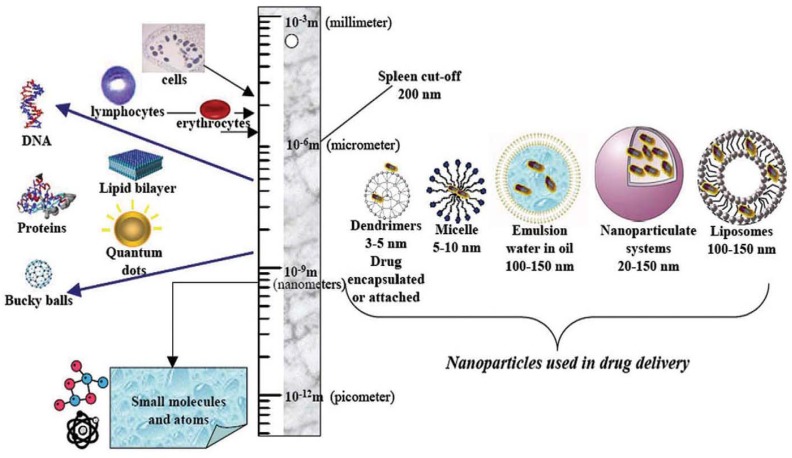
Nanoscale biomaterials and nanoparticle systems for drug delivery (adapted with permission from reference [5]).

Magnetic micro- and nanoparticles with biofunctional coatings are finding increasing use in fields such as microbiology, biomedicine and biotechnology where they are used to label, transport and separate biomaterials, and to deliver therapeutic drugs to a target tissue [1,2,3,4,5,6,7,8]. Magnetic particles, as shown in Figure 1, are well suited for bioapplications for several reasons. First, they are nontoxic and well-tolerated by living organisms when properly synthesized and functionalized [8]. Second, they can be synthesized in sizes that range from a few nanometers to tens of nanometers with a narrow size distribution. This makes them ideal for probing and manipulating bioparticles and biosystems such as proteins (5−50 nm), viruses (20−450 nm), genes (2 nm wide and 10−100 nm long), or whole cells (10–100 μm) (Figure 2) [2,5]. In this regard, it is important to note that submicron or micron-sized magnetic particles are also widely used for bioapplications (Figure 1b). Third, magnetic nanoparticles can be custom-tailored with appropriate surface treatments that enhance biocompatibility and enable coating with affinity biomolecules for highly specific binding with a target biomaterial. Fourth, magnetic nanoparticles exhibit superparamagnetic behavior, *i.e.,* they are easily magnetized by an applied magnetic field, but revert back to an unmagnetized state once the field is removed. Thus, they experience a magnetic force when subjected to a local field gradient, and they can be used to separate or immobilize magnetically labeled biomaterials from a carrier fluid using an external magnetic field. Significantly, the relatively low permeability of an aqueous carrier fluid enables efficient magnet coupling to an immersed magnetically labeled biomaterial. Moreover, the low intrinsic magnetic susceptibility of most biomaterials provides substantial contrast between labeled and unlabeled material, which enables a high degree of selectivity and detection. Magnetic labeling has advantages over conventional fluorescence and chemiluminescence based biolabels. Notably, small samples of magnetically labeled material can be detected using ultra-sensitive ferromagnetic “spin valve” sensors, which can be integrated into microfluidic-based diagnostic systems.

**Figure 3:**
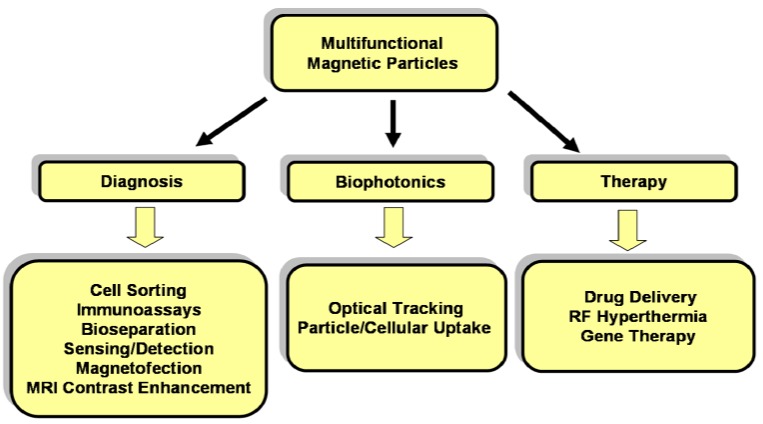
Bioapplications of magnetic particles.

Magnetic particles have additional advantages and uses that are not directly related to biotransport. Specifically, they can be designed to absorb energy at a resonant frequency from a time-varying magnetic field, which enables their use for therapeutic hyperthermia of tumors. Specifically, in RF hyperthermia magnetic nanoparticles are directed to malignant tissue and then irradiated with an AC magnetic field of sufficient magnitude and duration to heat the tissue to 42 °C for 30 min or more, which is sufficient to destroy the tissue [9]. Studies demonstrate that RF hyperthermia could be used as an alternate or an adjuvant to other cancer therapies [10,11]. Magnetic nanoparticles are also used for bioimaging; both optically, using surface-bound fluorofores for biophotonic applications [12,13,14,15,16,17], and magnetically where they serve as contrast agents for enhanced MRI. Common bioapplications of magnetic particles are listed in Figure 3.

In this article we review the use of magnetic nanoparticles as transport agents for various bioapplications. We begin with a brief summary of the preparation and properties of magnetic nanoparticles. This is followed by a detailed discussion of the physics and equations governing magnetic particle transport in a viscous medium. We discuss two different transport models: a classical Newtonian model for predicting the motion of individual particles, and a drift-diffusion model for predicting the behavior of a concentration of nanoparticles, which accounts for the effects of Brownian motion. Next, we review specific biotransport applications including magnetic bioseparation, drug delivery and magnetofection. We demonstrate the transport models *via* application to these processes. We conclude the review with an outlook for future prospects in this field.

## 2. Magnetic Nanoparticles

Magnetic nanoparticles typically range from 1–100 nm in diameter. However, larger particles, several hundred nanometers in diameter or even micron-sized, can be fabricated by encapsulating magnetic nanoparticles in organic (e.g., polymeric) or inorganic materials as shown in Figure 1b. Methods for synthesizing magnetic nanoparticles have evolved over several decades, and new methods continue to be developed and refined. There are two basic approaches to nanoparticle synthesis: bottom-up and top-down. In a bottom-up approach, elemental building blocks such as atoms, molecules or clusters are assembled into nanoparticles. This approach relies on the energetics of the process to guide the assembly. Examples of bottom-up chemical methods include coprecipitation, sonochemical reactions, sol-gel synthesis, microemulsions, hydrothermal reactions and hydrolysis and thermolysis of precursors [18,19,20,21]. The top-down approach involves the reduction of larger scale matter to desired nanoscale structures, and is generally subtractive in nature. Top-down methods include photolithography, mechanical machining/polishing, laser beam and electron beam processing, and electrochemical removal.

The most commonly used methods for preparing magnetic nanoparticles involve some form of bottom-up chemical approach. Such methods are routinely used to prepare particles of different materials including oxides such as magnetite Fe_3_O_4_ and maghemite γ-Fe_2_O_3_, pure metals such as Fe, Ni and Co, ferrites of the form *Me*O**∙**Fe_2_O_3_ , where *Me* = Mg, Zn, Mn, Ni, Co, ...), and alloys such as CoPt_3_ and FePt [18,19]. It should be noted that the most widely used magnetic nanoparticles are the oxides, Fe_3_O_4_ and γ-Fe_2_O_3_.

### 2.1. Properties of Magnetic Nanoparticles

The properties of a magnetic nanoparticle are typically very different from those of the constituent bulk material. Nanoscale properties can be strong functions of the physical attributes of the nanoparticle including its size, shape, chemical composition, crystal structure and morphology, as well as myriad surface effects. The magnetic response of noninteracting nanoparticles depends on their physical attributes as well as environmental factors such as the ambient temperature, the applied field, and the manner in which the temperature and field are varied. One of the most significant attributes that determines the magnetic state of a particle is its size. We consider four different states: multidomain (MD), pseudo-single domain (PSD), single domain (SD) and superparamagnetic (SP), which are ordered relative to the particle size to which they apply (*i.e.,* larger to smaller).

### 2.2. Multidomain Particles

**Figure 4:**
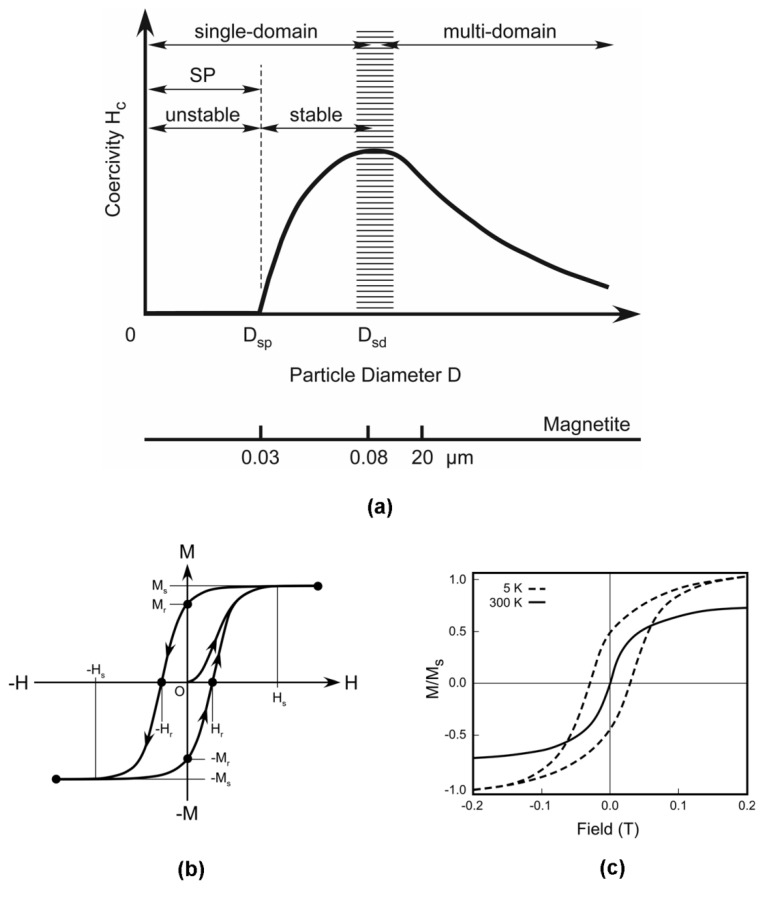
Properties of magnetic particles: (a) coercivity *versus* particle diameter, (b) magnetization curve for multidomain particles, (c) magnetization curves for superparamagnetic particles (superparamagnetic at room temperature 300 K, but not below the blocking temperature 5 K).

The MD state occurs in bulk materials. Specifically, a macroscopic specimen consists of a multitude of domains, wherein regions of uniform magnetization are separated by domain walls. Domains form in such a way so as to minimize the total energy of a specimen. The variables governing domain formation include the exchange and magnetocrystalline energies, as well as the saturation magnetization. These, in turn, depend on material composition and temperature. Thus, different materials have different domain state size dependence, and the domain state of a given specimen will vary with temperature. Domains vary in size, shape, and orientation, and the bulk magnetization of a specimen is defined by the collective behavior of all its domains. Field induced magnetization reversal in MD particles occurs *via* domain wall nucleation and motion. Since the energy required for these processes is relatively low, MD particles tend to have a relatively low remanent magnetization $M_{r}$ and coercivity $H_{c}$ (Figure 4a). A typical magnetization curve (*M versus H*) for a MD specimen is shown in Figure 4b, which shows the saturation magnetization $M_{s}$ and saturating field $H_{s}$.

As the size of a MD specimen decreases, it transitions to a PSD state, with properties and behavior that are intermediate between the MD and SD states. While the magnetic behavior of PSD particles is not fully understood, they are thought to have relatively few domains, and tend to have MD-like low coercivity, but SD-like high-remanence. The PSD state occurs in Fe_3_O_4_ (magnetite) particles ranging in size from 0.1–20 μm (Figure 4a).

### 2.3. Single Domain Particles

As the size of a magnetic specimen continues to decrease there is a critical volume below which the energetics favor a SD state. The transition occurs when the magnetostatic energy $E_{ms}$ of the particle becomes comparable to the energy $E_{wall}$ required to form a domain wall within the particle. The critical diameter $D_{sd}$ for this transition for a spherical particle depends on the material, and is typically in the range of a few tens of nanometers: e.g., $D_{sd}$ = 15 nm, 55 nm and 128 nm for Fe, Ni, and Fe_3_O_4_, respectively. In SD particles, below the characteristic Curie temperature of the material, the individual spins are ferromagnetically coupled and uniformly aligned, and the particle becomes spontaneously magnetized with a relatively large magnetic moment, typically 10³ to 10^5^ $\mu_{B}$, where $\mu_{B}$ = 9.274 × 10^–24^ J/T is the Bohr magneton. In the absence of an applied field, the magnetization of the particle lies along a preferred “easy axis”, which is defined by magnetic anisotropies that can be due to many factors including the particle's shape, chemical composition, crystalline structure and residual strain, as well as various surface effects. The magnetization will remain in this state as long as the anisotropy energy $E_{a}$ = $K_{eff}V$ is large relative to the thermal energy k T, where $K_{eff}$ and *V* are the effective anisotropy constant and particle volume, respectively, k = 1.38 ×10^23^ m^2^∙kg∙s^−2^∙K^−1^ is Boltzmann's constant, and *T* is temperature in Kelvin. Field induced magnetization reversal in such particles occurs *via* spin rotation against the anisotropy "force" (there are no domain walls to move). Consequently, they exhibit a larger coercivity than MD materials because it is energetically more difficult to overcome the anisotropy energy than it is to nucleate or move a domain wall. The magnetization curve takes the form of a square hysteresis loop, and thus SD particles are magnetically hard, having both a high coercivity and high remanence (Figure 4a).

### 2.4. Superparamagnetic Particles

As the volume of a SD particle decreases, its anisotropy energy eventually reduces to a level where it is comparable to the thermal energy. At this point the particle has a diameter $D_{sp}$ and the magnetization can spontaneously flip from one easy direction to another, even when there is no applied field. The particle is said to be in a superparamagnetic (SP) state (Figure 4a and Figure 4c, T = 300 K curve). The thermally-induced frequency f of switching between the different easy axes directions is given by

(1) $$f=f_{0}\text{exp}\left(-\frac{E_{b}}{k_{B}T}\right)\;,$$

where $f_{0}$ is an attempt frequency, which is typically taken to be 10^9^ Hz, and $E_{b}$ is the total energy barrier to switching ( $E_{b}=E_{a}$ if there is no applied field). If an ensemble of SP particles are subjected to changes in the applied field or temperature, the magnetization will approach an equilibrium value in a period τ=1/f. which is called the relaxation time, first derived by Neel, *i.e.,*

(2) $$\tau =\tau_{0}\text{exp}\left(\frac{K_{eff}V}{k_{B}T}\right)\;$$

where $\tau_{0}=10^{-9}$s. If τ is shorter than the time required to measure the magnetization of the nanoparticles, they are said to be in a superparamagnetic state. On the other hand, if τ is longer than the measurement time, the nanoparticles are said to be "blocked". Below the blocking temperature, the particles possess coercivity and remanent magnetization, and the magnetization as a function of temperature and magnetic field are similar to those of bulk ferromagnetic materials below the Curie temperature as shown in Figure 4c (T = 5 K curve). The temperature that defines the transition between the blocked and unblocked states is the called the blocking temperature $T_{B}$, if $T<T_{B}$ the particle is blocked, whereas if $T>T_{B}$ it is unblocked.

The magnetic response of an ensemble of identical noninteracting SP particles is essentially the same as that of a paramagnet except that the magnetic moment is not that of a single atom, but rather of SD particle that contains 10^5^ atoms. Thus, the susceptibility is much higher than that of a common paramagnet, and hence the term superparamagnetic. The magnetization of an ensemble of paramagnetic particles is given by

(3) $$M=n\mu L\left(\frac{\mu H_{a}}{kT}\right)$$

where L(y)=coth(y)−1/y is the Langevin function, n is the number of particles per unit volume (nμ is the saturation magnetization of the assembly), and $\mu =M_{s}\;V$ is the magnetic moment per particle, which is a strong function of the particle size. The magnetization is zero if there is no applied field, the coercivity and remanent magnetization are zero, and there is a very high field saturation as shown in Figure 4c (T = 300 K).

## 3. Magnetic Particle Transport

Magnetophoresis involves the manipulation of colloidal magnetic particles in a viscous medium using an applied magnetic field. The motion of a magnetic particle in a carrier fluid is governed by several factors including (a) the magnetic force, (b) viscous drag, (c) particle/fluid interactions (perturbations to the flow field), (e) gravity/buoyancy, (g) thermal kinetics (Brownian motion), and (h) interparticle effects including; (i) magnetic dipole-dipole interactions, (ii) electric double-layer interactions, and (iii) van der Walls force. In this section, we review the basic equations governing magnetophoretic particle transport. To simplify the analysis, we consider particles in low concentration and neglect particle/fluid interactions and interparticle effects. Particle/fluid interactions can be taken into account by solving for the flow field and particle trajectories numerically, applying appropriate fluidic boundary conditions of the surface of the particles during each time step. Similarly, interparticle (e.g., dipole-dipole) interactions can be included in the equations of motion and calculated during each time step. However, the details of these computations are complicated and beyond the scope of this paper.

In many applications, particle diffusion due to Brownian motion can also be neglected, which further simplifies the analysis. This is true when the applied (e.g., magnetic) forces on the particle dominate the force it experiences due to random collisions with the fluid molecules. However, for a given application, it is not always clear whether or not Brownian motion can be ignored, as the applied force can vary substantially throughout the region of interest. Nevertheless, the effects of Brownian motion are usually ignored for particles that are greater than a few tens of nanometers in diameter as discussed below.

**Figure 5:**
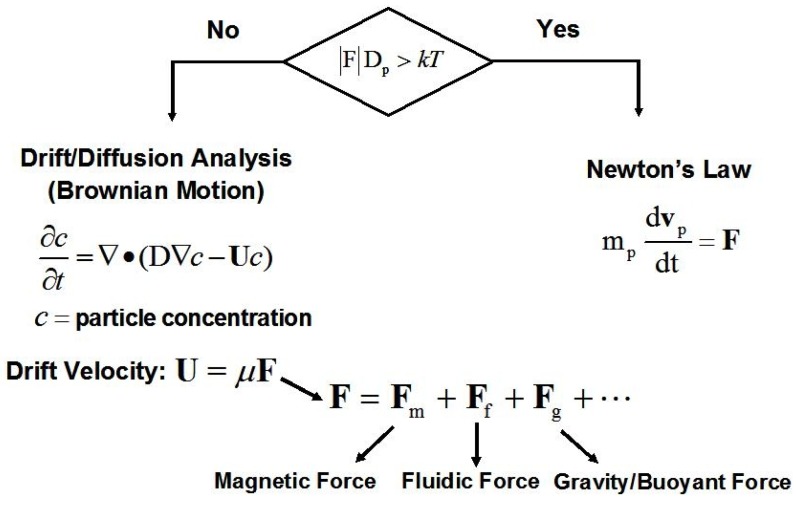
Magnetic particle transport analysis.

We present two different models for predicting magnetic particle transport in a carrier fluid, each governing a different regime of transport as shown in Figure 5. In the first model, we neglect Brownian motion and use classical Newtonian physics to predict the motion of individual particles, submicron-sized or larger. In the second model, we account for Brownian motion by solving a drift-diffusion equation for the behavior of a concentration of non-interacting magnetic nanoparticles.

### 3.1. Newtonian Particle Transport

We consider the motion of a spherical magnetic particle in a viscous carrier fluid under the influence of an applied field. We restrict our attention to slow flow regimes where the magnetic and viscous drag forces dominate, and we neglect Brownian motion. The particle has a density $\rho_{p}$, radius $R_{p}$, volume $V_{p}=\frac{4}{3}\pi R_{p}^{3}$, and mass $m_{p}=\rho_{p}V_{p}$. We use classical Newtonian dynamics to study particle motion [22],

(4) $$m_{p}\frac{du_{p}}{dt}=F_{m}+F_{f}+F_{g}$$

where $u_{p}$ is the velocity of the particle, and $F_{m}$, $F_{f}$, and $F_{g}$ are the magnetic, fluidic, and gravitational forces, respectively. The inertial term $m_{p}\frac{du_{p}}{dt}$ is often ignored for submicron particles as their mass is negligible. When this is the case, the particle motion is based on a simple force balance

(5) $$F_{m}+F_{f}+F_{g}=0$$

This equation can be solved for the instantaneous particle velocity $u_{p}$ given an expression for the fluidic force. Specifically, the fluidic force is obtained using Stokes’ law for the viscous drag on a sphere as described below,

(6) $$F_{f}=-6\pi \eta R_{hyd,p}\;(u_{p}-u_{f}),$$

where $R_{hyd,p}$ is the effective hydrodynamic radius of the particle, and η and $u_{f}$ are the viscosity and velocity of the fluid, respectively. It is important to note that $R_{hyd,p}$ is greater than the physical radius of the particle as surface-bound materials contribute to the viscous drag. Substitute Equation (6) into Equation (5) and obtain $u_{p}=u_{f}+\gamma \left(F_{m}+F_{g}\right)$ or

(7) $$\frac{dx_{p}}{dt}=u_{f}+\gamma \left(F_{m}+F_{g}\right)$$

where $\gamma =1/(6\pi \eta R_{hyd,\;p})$ is the mobility of the particle. We use either Equation (4) or Equation (7) to predict the motion of a particle depending on the significance of the particle inertia. This can be estimated as follows. Consider Equation (4) in one dimension when the only force on the particle is fluidic *i.e.*, $F_{f}$ given by Equation (6), and no magnetic or gravitational force present,

(8) $$m_{p}\frac{du_{p}}{dt}=6\pi \mu R_{p}\left(u_{f}-u_{p}\right)$$

This equation has a solution of the form (with $u_{p}(0)=0$)

(9) $$u_{p}(t)=u_{f}\left(1-e^{-\frac{t}{\tau }}\right)$$

where $\tau =\frac{m_{p}}{6\pi \mu R_{p}}=\frac{2\rho_{p}R_{p}^{2}}{9\mu }$ is a time constant for the particle to obtain its terminal velocity, *i.e.,* the local velocity of the fluid. For Fe_3_O_4_ particles in water with a radius between 1 nm–100 nm, *τ* ranges from 1.11 ps – 11.1 ns, respectively. Thus, for many applications the nanoparticles acquire their terminal velocity on a time scale that is much shorter than the overall transport time through a system. When this is the case, we ignore particle inertia and solve Equation (7) for the motion of the particle.

### 3.2. Drift-Diffusion Transport

As noted above, Equation (4) does not take into account Brownian motion, which can influence particle motion when the particle diameter $D_{p}$ is sufficiently small. We estimate this diameter using the following criterion [23],

(10) $$\left|F\right|D_{p}\leq kT$$

where |F| is the magnitude of the applied force acting on the particle. This condition implies that Brownian motion needs to be taken into account when the energy exerted by the applied force in moving the particle a distance equal to its diameter is less than or comparable to thermal energy *kT*. In order to apply Equation (10), one needs to estimate |F|. If a field source is specified, and the magnetic force is the dominant force, then one can estimate |F| for a given particle over the region of interest. Gerber *et al.* have studied the capture of Fe_3_O_4_ particles in water using a single magnetic wire, and have estimated the critical particle diameter for their application to be $D_{c,p}\equiv kT/\left|F\right|$ = 40 nm, *i.e.*, |F|= 0.1 pN [23]. For particles with a diameter below $D_{c,p}$ (which is application dependent) one solves a drift-diffusion equation for the particle volume concentration c, rather than the Newtonian equation for the trajectory of a single particle. Specifically, c is governed by the following equation [23,24,25],

(11) $$\frac{\partial c}{\partial t}+\nabla •J=0$$

where $J=J_{D}+J_{A}$ is the total flux of particles, which includes a contribution $J_{D}=-D\nabla c$ due to diffusion, and a contribution $J_{A}=U\;c$ due to the drift of the particles under the influence of applied forces. The diffusion coefficient D is given by the Nernst-Einstein relation D=γkT, where $\gamma =1/(6\pi \eta R_{hyd,p})$ is the mobility of the particle. The drift velocity U in $J_{A}$ is obtained from Equation (4) in the limit of negligible inertia ($m_{p}\frac{du_{p}}{dt}\to 0$), *i.e.,* by setting $F_{m}+F_{f}+F_{g}=0$. Specifically, from Equation (7) we find that U=γF, where $F=\frac{u_{f}}{\gamma }+F_{m}+F_{g}$. Note that if the Stokes’ drag is the only force, then $U=u_{f}$**.** In order to predict either Newtonian or Drift Diffusion transport, one needs expressions for the various forces on the particles. These are derived in the flowing sections.

### 3.3. The Magnetic Force

We model the magnetic force using an “effective” dipole moment approach wherein the magnetized particle is replaced by an “equivalent” point dipole [26]. The magnetic force on the dipole (and hence on the particle) is given by,

(12) $$F_{m}=\mu_{f}\left(m_{p,eff}•\nabla \right)H_{a}$$

where $\mu_{f}$ is the permeability of the transport fluid, **m***_p,eff_* is the“effective” dipole moment of the particle, and $H_{a}$ is the applied magnetic field intensity at the center of the particle, where the equivalent point dipole is located. The dipole approximation has been used for decades to compute the force on submicron magnetic particles. The validation of this approximation has recently been confirmed *via* particle trajectory measurements in a microfluidic system [27].

In order to determine the magnetic force, one needs to know the field distribution and the magnetic response (*i.e., M vs*. *H*) of the particle. The magnetic field distribution can be determined once the field source and system materials are specified. In many bioapplications, the materials (e.g., carrier fluid, fluidic system and biomaterial) are essentially nonmagnetic and the field can be obtained in an analytical form. Otherwise, it can be determined using numerical techniques such as finite element analysis.

In order to determine the effective moment $m_{p,eff}$ we need a model for the magnetization of the particle. A magnetization model that takes into account self-demagnetization and magnetic saturation has been developed, and is briefly summarized here [24,26,28,29]. Consider a magnetic nanoparticle with a radius $R_{p}$ and volume $V_{p}$ in the presence of an applied magnetic field $H_{a}$. Assume that particle is uniformly magnetized, and that the magnetization is a linear function of the field intensity up to saturation, at which point it remains constant at a value $M_{s}$. Specifically, below saturation,

(13) $$M_{p}=\chi_{p}H_{in}$$

where $\chi_{p}=\mu_{p}/\mu_{0}-1$ is the susceptibility of the particle, and $\mu_{p}$ is its permeability. The field inside the particle is a superposition of the applied field and the internal demagnetization field, $H_{in}=H_{a}-H_{demag}$. $H_{demag}$ is the self-demagnetization field that accounts for the magnetization of the particle, *i.e.,* its magnetic “surface charge”. $H_{demag}=M/3$ for a uniformly magnetized spherical particle with magnetization M in free-space (p. 25 of [30]).

If the particle is suspended in a magnetically linear fluid of permeability $\mu_{f}$, the force on it in an applied field $H_{a}$ is [26]

(14) $$F_{m}=\mu_{f}V_{p}\frac{3\left(\chi_{p}-\chi_{f}\right)}{\left[\left(\chi_{p}-\chi_{f}\right)\;+\;3\left(\chi_{f}+1\right)\right]}\left(H_{a}•\nabla \right)H_{a}$$

where $\chi_{f}$ is the susceptibility of the carrier fluid. For most bioapplications $\left|\chi_{f}\right|<<1$ ($\mu_{f}\approx \mu_{0}$), in which case Equation (14) reduces to

(15) $$F_{m}=\mu_{0}V_{p}\frac{3\left(\chi_{p}-\chi_{f}\right)}{\left(\chi_{p}-\chi_{f}\right)+3}\left(H_{a}•\nabla \right)H_{a}.$$

Under this assumption we also find that

(16) $$M_{p}=\frac{3\left(\chi_{p}-\chi_{f}\right)}{\left(\chi_{p}-\chi_{f}\right)+3}H_{a}$$

Equation (16) applies below saturation. However, when the particle is saturated, $M_{p}=M_{sp}$. We account for both conditions by expressing the magnetization in terms of the applied field as follows,

(17) $$M_{p}=f(H_{a})H_{a}$$

where

(18) $$f(H_{a})=\{\begin{array}{cc} \frac{3\left(\chi_{p}-\chi_{f}\right)}{\left(\chi_{p}-\chi_{f}\right)+3} & H_{a}<\left(\frac{\left(\chi_{p}-\chi_{f}\right)+3}{3\left(\chi_{p}-\chi_{f}\right)}\right)M_{sp}\\ M_{sp}/H_{a} & H_{a}\geq \left(\frac{\left(\chi_{p}-\chi_{f}\right)+3}{3\left(\chi_{p}-\chi_{f}\right)}\right)M_{sp} \end{array}$$

and $H_{a}=\left|H_{a}\right|$. The dipole moment is $m_{p,eff}=V_{p}M_{p}$ or $m_{p,eff}=V_{p}f(H_{a})H_{a}$.

It is instructive to evaluate Equation (12) using the expression for $m_{p,eff}$. Consider a two-dimensional system in which the field source is assumed to be of infinite extent in the *z*-direction, and therefore the *z*-components of the magnetic field and force are zero. Under this assumption we obtain,

(19) $${\text{H}}_{a}(x,y)=H_{ax}(x,y)\hat{\text{x}}+H_{ay}(x,y)\hat{\text{y}}$$

and

(20) $${\text{F}}_{m}(x,y)=F_{mx}(x,y)\hat{\text{x}}+F_{my}(x,y)\hat{\text{y}}$$

where

(21) $$F_{mx}(x,y)=\mu_{0}V_{p}\;f(H_{a})\left[H_{ax}(x,y)\frac{\partial H_{ax}(x,y)}{\partial x}+H_{ay}(x,y)\frac{\partial H_{ax}(x,y)}{\partial y}\right]$$

and

(22) $$F_{my}(x,y)=\mu_{0}V_{p}\;\;f(H_{a})\left[H_{ax}(x,y)\frac{\partial H_{ay}(x,y)}{\partial x}+H_{ay}(x,y)\frac{\partial H_{ay}(x,y)}{\partial y}\right]$$

We can evaluate $F_{m}(x,y)$ given an expression for the field distribution $H_{a}(x,y)$. Analytical expressions for the field distributions of common magnetic sources (*i.e.*, cylindrical and rectangular rare-earth magnets, and rectangular current carrying conductors) can be found in the literature [22,23,24,25,26,28,29,30,31].

### 3.4. The Fluidic Force

The fluidic force is predicted using Stokes’ law for the viscous drag on a sphere,

(23) $$F_{f}=-6\pi \eta R_{hyd,p}\;f_{D_{p}}(u_{p}-u_{f}),$$

where $R_{hyd,p}$ is the hydrodynamic radius, and η and $u_{f}$ are the viscosity and velocity of the fluid, respectively. The term $f_{D_{p}}$ is a drag coefficient that accounts for the influence of a solid wall in the vicinity of the moving particle [32]. If the particle is far from the wall, $f_{D_{p}}$ = 1 and Equation (23) reduces to the usual Stokes’ drag formula, which strictly applies to a single isolated particle in an infinite uniform flow field. However, for most applications the flow field is not uniform but rather varies throughout the fluidic system (e.g., laminar flow through a microchannel). Nevertheless, the particle diameter is typically much smaller than the dimensions of the fluidic system, and thus the fluid velocity is usually relatively constant across the particle. Thus, we use Equation (23) to estimate the viscous drag force on a particle at a given time, using the particle velocity at that time, and the fluid velocity at the position of the particle at that time.

### 3.5. The Gravitational Force

The gravitational force takes into account buoyancy and is given by

(24) $$F_{g}=-V_{p}(\rho_{p}-\rho_{f})g\;$$

where $F_{g}=\left|F_{g}\right|$ is the magnitude of the force, $\rho_{p}$ and $\rho_{f}$ are the densities of the particle and fluid, respectively, and $g=9.8\;\text{m}/{\text{s}}^{2}$ is the acceleration due to gravity. The force is often ignored when analyzing the motion of submicron particles, as it is usually much weaker than the magnetic force [22,28]. In order to verify this, it is instructive to evaluate $F_{g}$ for a one micron ($R_{p}=0.5$ μm) Fe_3_O_4_ particle in water ($\rho_{p}=5000$ kg/m^3^, $\rho_{f}=1000$ kg/m^3^). We evaluate the force due to the particle mass $F_{mass}$ and buoyancy $F_{b}$ separately, $F_{mass}=\rho_{p}\frac{4}{3}\pi R_{p}^{3}g$ and $F_{b}=\rho_{f}\frac{4}{3}\pi R_{p}^{3}g$, and obtain $F_{mass}$ = 2.618 × 10^-2^ pN, and $F_{b}$ = 0.523 × 10^-2^ pN. These forces are typically an order of magnitude smaller than the applied magnetic force.

## 4. Bioapplications

As noted in the introduction, the use of magnetic particles for bioapplications is proliferating. Current applications include magnetic drug and gene delivery, RF hyperthermia [9], cell sorting, [33,34], bioassays [6], magnetofection [35,36], magnetic twisting cytometry [37], MRI [9], biosensing and bioseparation [38] (Figure 3). In this section we discuss biofunctionalization of magnetic particles, and specific applications in which the particles are used to transport, immobilize or separate biomaterial.

### 4.1. Biofunctionalization

**Figure 6:**
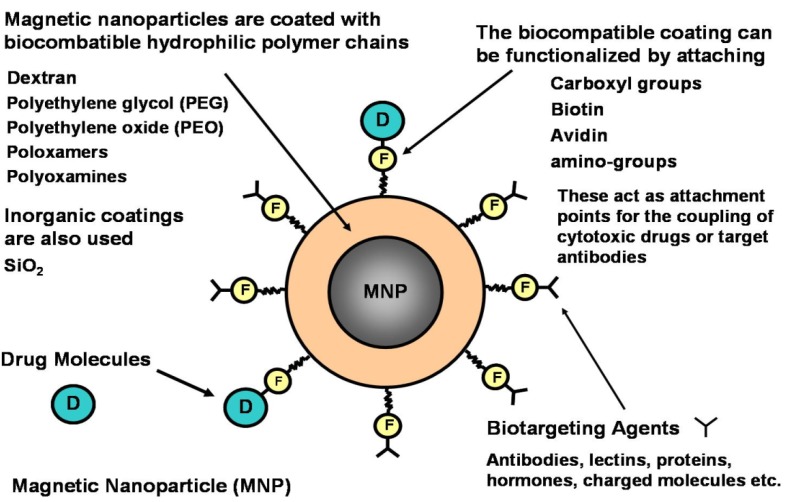
Biofunctional magnetic nanoparticles.

Magnetic micro- and nanoparticles need to be functionalized (treated with appropriate surface coatings) to be of use in bioapplications (Figure 6). For such applications there are several issues to consider: colloidal stability, biocompatibility and biotargeting [3,4,6,18]. Colloidal stability implies that the particles do not aggregate in a carrier fluid. Nanoparticles in a colloidal dispersion undergo Brownian motion and frequently collide with one another. The dispersion will not be stable if the particles aggregate upon collision. Attractive interactions such as Van der Waals, electrostatic, and magnetic dipole-dipole forces can cause irreversible aggregation. These forces need to be countered if the colloid is to remain stable. To this end, the particles are surface-modified to create electrostatic or steric repulsive forces. Electrostatic stabilization involves coating the nanoparticles with materials that produce an electrical double-layer on the surface. The nanoparticles are stabilized by mutual electrostatic repulsion, which is substantial because of their large surface area-to-volume ratio. Steric stabilization is achieved by coating the nanoparticles with surfactants "surface acting agents" or polymers. Surfactants are typically organic compounds that are amphiphilic, *i.e.*, they contain both hydrophilic groups (heads) pointing outward, and hydrophobic groups (tails) pointing inward. Surfactants for magnetic nanoparticles typically contain functional groups such as carboxylic acids, phosphates, and sulfates. Common polymeric stabilization coatings include dextran, polyethylene glycol (PEG), and polyvinyl alcohol (PVA), which also aid in biocompatibility [6].

Biocompatibility of a particle implies that it will function within a complex biosystem with minimal undesired interactions (e.g., nonspecific adsorption events) that would otherwise degrade its functionality, e.g., ability to recognize or bind to a target biomaterial. For example, for *in vivo* applications such as drug targeting, magnetic particles move through the vascular system and must be treated with an appropriate coating to resist opsonization [5]. In opsonization, opsonins (plasma proteins including antibodies) bind to the particles and attract phagocytes, which remove particles from the vascular system *via* phagocytosis with subsequent accumulation in organs of the reticuloendothelial system (RES) such as the spleen and liver. The amount and types of plasma proteins that bind to the particles is determined by their physicochemical surface characteristics. Commonly used biocompatible coatings include dextran, PEG and PVA, as previously noted. Inorganic materials such as gold and silica are also used as biocompatible coating materials.

Biotargeting functionality is added to magnetic particles by coating their surface with appropriate biotargeting agents such as antibodies, proteins and charged molecules *etc*. (Figure 6). For example, magnetic nanoparticles with specific surface-bound antibodies can selectively bind to target cells that have the antibody receptors [33,34,39]. Magnetic particles coated with immunospecific agents have been successfully bound to red blood cells, lung cancer cells, bacteria, and urological cancer cells, among others. The advantage of biotargeting is that it facilitates the selective delivery of particle-bound therapeutic agents to a desired “target” tissue. The idea is that magnetic particles with surface-bound therapeutic agents are directed over a relatively longer length scale (cm) to a target tissue using an applied magnetic field, and once in proximity to the tissue the particle-bound biotargeting agents promote selective binding to the tissue on the cellular level.

### 4.2. Bioseparation and Bioassays

**Figure 7:**
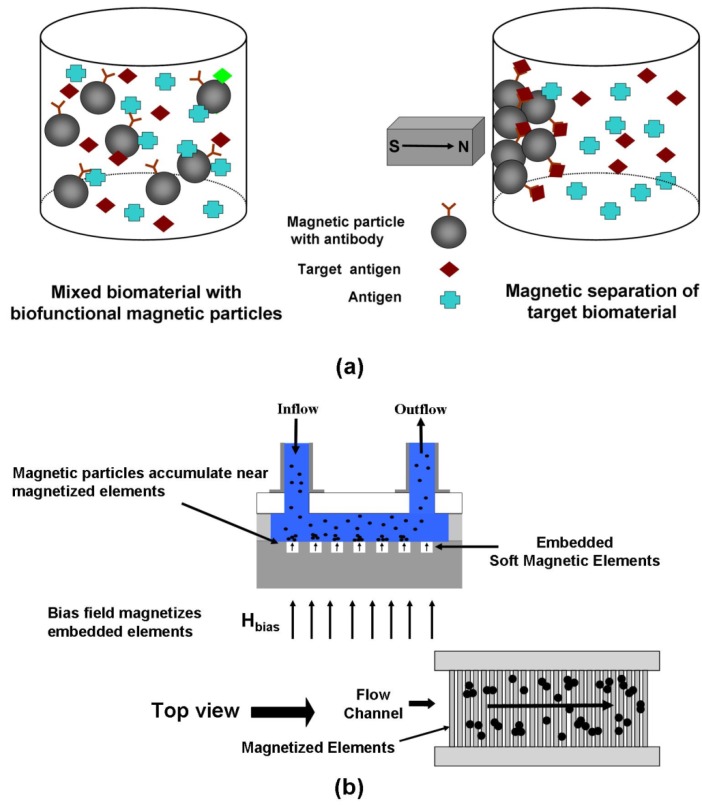
Magnetic bioseparation: (a) illustration of bioseparation process, (b) magnetophoretic microsystem for bioseparation.

The ability to separate, sort or immobilize biomaterials such as proteins, enzymes, nucleic acids (e.g. RNA and DNA) or whole cells in their native environment is fundamental to the detection and analysis of such entities [1,2,6,38,40,41,42]. Magnetic separation or immobilization of a biomaterial involves the use of magnetic micro- or nanoparticles with surface-immobilized affinity ligands that are selected to bind with the target biomaterial. Magnetic particles can be used to separate the target biomaterial from a low concentration sample using an applied field (Figure 7a). Once separated, the biomaterial can be released into an appropriate medium in a sufficiently high concentration to enable a desired analysis by removing the field. Magnetic separation is used for sample preparation in various applications including high throughput genome isolation for sequencing or polymerase chain reactions to carry out genotyping. The separation of nucleic acids from their native complex mixtures is required to enable processes such as sequencing, amplification, hybridization and detection. Magnetic particles have been used for various immuoassays including fluorescence or enzyme immunoassays [43].

Magnetic bioseparation is usually implemented using either a direct or indirect approach [44]. In the more common direct approach the magnetic particles are functionalized with specific ligands that will bind with the target biomaterial. These particles are mixed with a solution containing the biomaterial, and the mixture is allowed to incubate until a sufficient amount of the target biomaterial binds to the particles. The magnetically labeled biomaterial is then separated from the solution using a magnetic separation system, and then re-released in higher concentration in an appropriate medium for further processing.

In the indirect approach, the target biomaterial is first incubated in solution with an affinity ligand (primary antibody), which is added in free form. After a sufficient amount of biomaterial binds to the primary antibody, magnetic particles with surface-bound secondary antibodies (antibodies against the primary antibodies) are introduced, and the mixture is allowed to incubate until a sufficient amount of the target biomaterial becomes magnetically labeled. The labeled material is then magnetically separated and re-released in higher concentration for further processing.

In conventional magnetic separation systems rare-earth magnets or electromagnets are used to produce a non-uniform field distribution throughout the separation region. When magnetic particles enter this region they experience a force that moves them towards areas of high field gradient where they can be captured as shown in Figure 7a. The particles have a high susceptibility and acquire a dipole moment in an external field, but quickly relax back to an unmagnetized state once the field is removed. Thus, when the field is removed the separated particles disperse into a desired solution for further processing.

Conventional magnetic separation systems have drawbacks in that they tend to be relatively large, costly, and complex, requiring significant energy to operate. Moreover, the accurate manipulation of microscopic particles in small sample volumes is awkward and time consuming in such systems, and the ability to precisely monitor the separation process is limited. However, advances in microsystem technology have led to the development of novel integrated magnetic bioseparation microsystems that are energy efficient and ideal for the analysis and monitoring of small samples [22,29,45,46,47,48,49,50,51,52,53,54].

**Figure 8:**
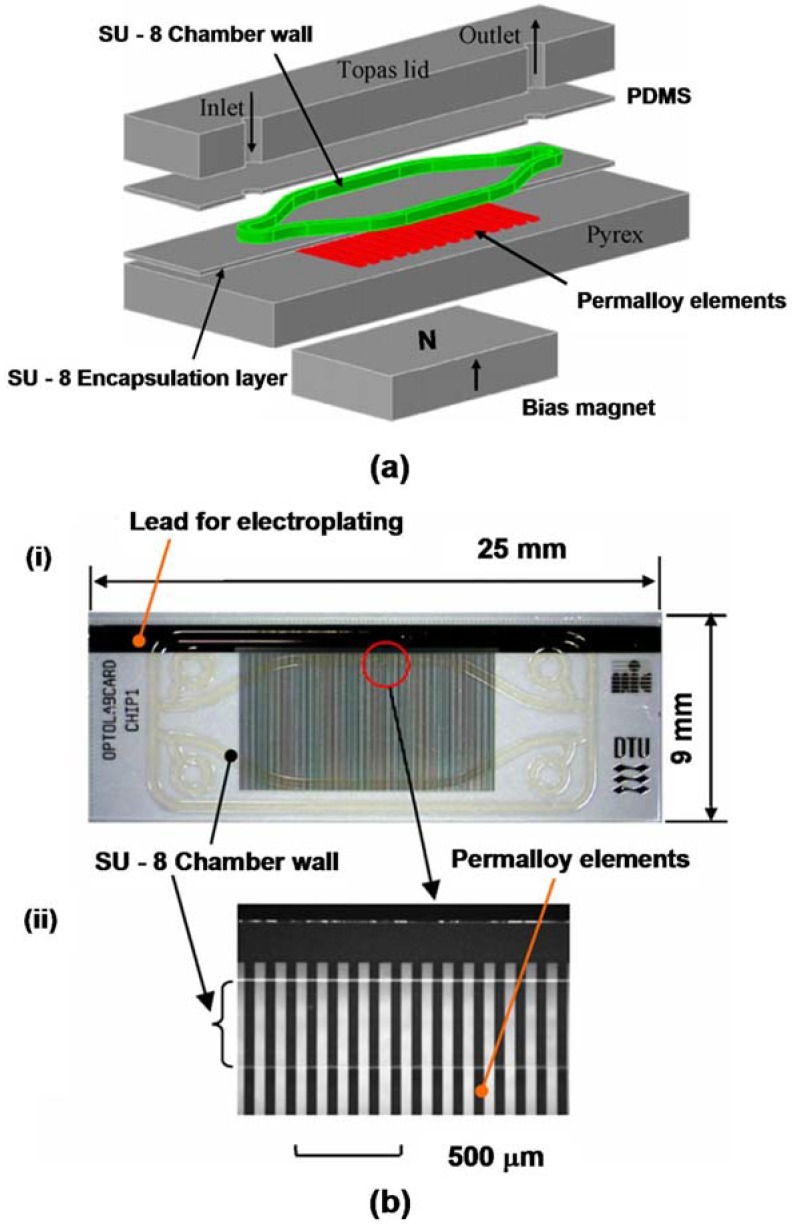
Passive magnetophoretic microsystem: (a) exploded view of the microsystem, (b) image of chip with electroplated permalloy elements and SU-8 fluidic chamber wall (adapted with permission from reference [53]).

**Figure 9:**
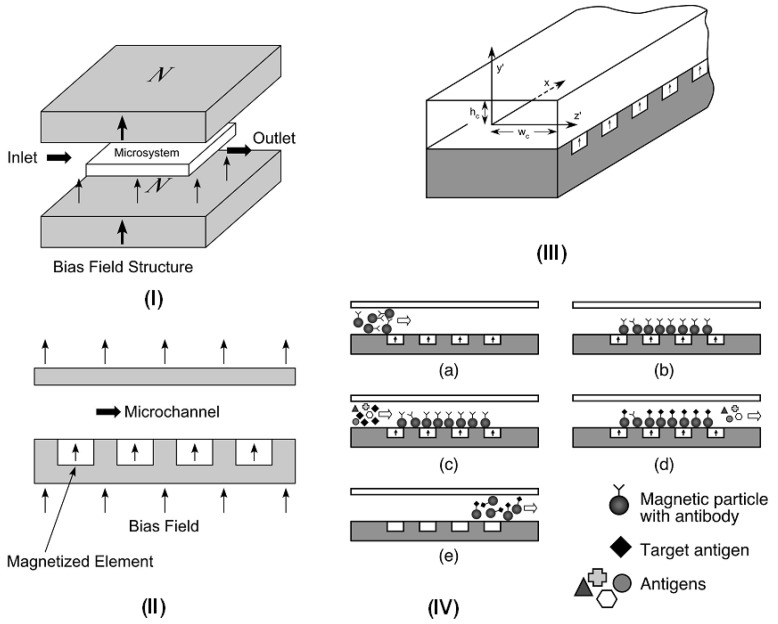
Magnetophoretic microsystem for bioseparation: (I) microsystem with bias field structure, (II) cross-section of the microsystem showing the microchannel, (III) geometry and reference frame for the microfluidic channel, (IV) cross-section of microsystem illustrating bioseparation sequence: (a) magnetic particles with surface-bound antibodies enter the microchannel with the bias field applied and the elements magnetized, (b) magnetized elements capture the particles, (c) target antigens are introduced into the microchannel, (d) target antigens become immobilized on captured magnetic particles, (e) the bias field is removed and the magnetic elements revert to an unmagnetized state releasing the separated material for further processing.

An example of a microsystem for magnetic bioseparation is shown in Figure 7b, Figure 8, and Figure 9. This system consists of an array of integrated soft-magnetic elements embedded in a nonmagnetic substrate beneath a microfluidic channel [53]. A bias field is used to magnetize the elements, which produce a field gradient that imparts a force to magnetic particles within the microchannel. This system has been studied using a Newtonian transport model [22,28]. The equations of motion for a magnetic nanoparticle traveling through the microchannel can be written in component form as follows,

(25) $$m_{p}\frac{du_{p,x}}{dt}=F_{mx}(x,y)-6\pi \eta R_{p}\left[u_{p,x}-\frac{3\;{\bar{u}}_{f}}{2}\left[1-{\left(\frac{y-(h+h_{c}+t_{b})}{h_{c}}\right)}^{2}\right]\right]$$

(26) $$m_{p}\frac{du_{p,y}}{dt}=F_{my}(x,y)-6\pi \eta R_{p}u_{p,y}$$

(27) $$u_{p,x}(t)=\frac{dx_{p}}{dt},\;u_{p,y}(t)=\frac{dy_{p}}{dt}.$$

where *h* and *h_c_* are the half heights of the magnet and fluidic channel, respectively, and *t_b_* is the thickness of the base of the channel (*i.e.*, the distance from the top of the magnetic elements to the lower edge of the fluid). Note that here we have assumed that the hydrodynamic radius of the particle is the same as its physical radius. Equations (25)-(27) constitute a coupled system of first-order ordinary differential equations (ODEs) that are solved subject to initial conditions for x(0), y(0), $u_{p,x}(0)$, and $u_{p,y}(0)$. These equations can be solved numerically using various techniques such as the Runge-Kutta method.

We demonstrate the use of Equations (25)-(27) *via* application to a microsystem with a fluid channel that is 200 μm high, 1 mm wide, and 10 mm long ($h_{c}$ = 100 μm, $w_{c}$ = 500 μm in Figure 9 III). The fluid is nonmagnetic ($\chi_{f}=0$), and has a viscosity and density equal to that of water, η=0.001 N∙s/m^2^, and $\rho_{f}=1000$ kg/m^3^∙ There are 10 permalloy (78% Ni 22% Fe) elements embedded immediately beneath the microchannel (p. 43 in [30]). Each element is 100 μm high, and 100 μm wide (h = 50 μm, w = 50 μm), and they are spaced 50 μm apart (edge to edge). The bias field is 0.25 T, which is provided by a single NdFeB magnet positioned beneath the microsystem. We assume that this field is sufficient to magnetize the permalloy elements to saturation, $M_{es}=8.6\times 10^{5}\;\text{A}/\text{m}$. We consider the transport of Fe_3_O_4_ particles that have the following properties: $R_{p}$ = 250 nm, $\rho_{p}=5000\;{\text{kg}/\text{m}}^{3}$, and *M_sp_* = 4.78 × 10^5^ A/m. We adopt a magnetization model for the particles that is consistent with Equation (18) when χ_p_ >> 1, *i.e.*,

(28) $$f(H_{a})=\{\begin{array}{cc} 3 & H_{a}<M_{sp}/3\\ M_{sp}/H_{a} & H_{a}\geq M_{sp}/3 \end{array}$$

**Figure 10:**
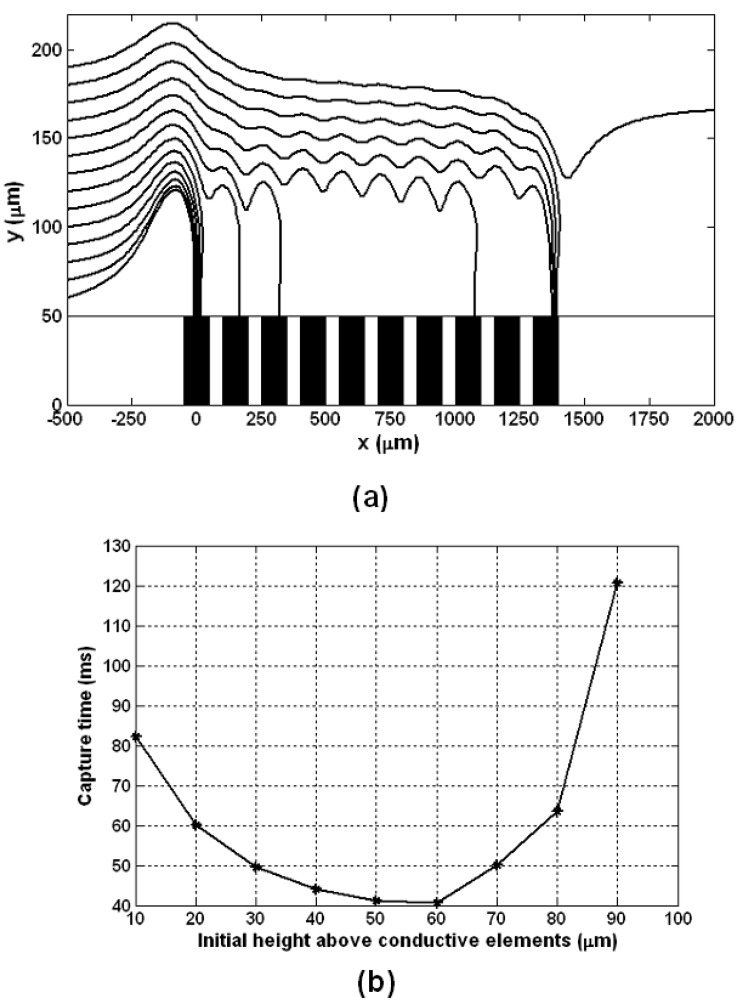
Bioseparation microsystem analysis: (a) Fe_3_O_4_ particle trajectory *versus* entry height above the magnetized elements, (b) particle capture time *versus* entry height above the magnetized elements.

We study the behavior of a particle as it moves through the microsystem. We assume that the particle enters the microchannel to the left of the first element at x(0)=−10w, and compute its trajectory as a function of its initial height above the magnetized elements: Δ*y* = 10 μm, 20 μm, …, 140 μm (*i.e.*, initial heights of *y*(0) = 60 μm, 70 μm, …, 190 μm). The average fluid velocity is ${\bar{u}}_{f}=10\;\text{mm}/\text{s},$ and the particle enters the channel with this velocity, $u_{p}(0)=10\;\text{mm}/\text{s}.$ The computed particle trajectories are shown in Figure 10a.

It is easy to identify each trajectory with its entry height as this is the y-intercept for the plot. According to the analysis, particles that enter the microchannel 0–130 μm above the magnetized elements (*i.e., y* = 50–180 μm) will be captured, but those entering at a greater height will pass through the system. The capture time (the time it takes for a particle to reach the bottom of the microchannel where it is held in place) is plotted as a function of the entry height in Figure 10b. This plot shows that particles entering at heights 0–90 μm above the elements will be captured within 130 ms.

Notice that the capture time is minimum for particles that enter the channel 50–60 μm above the elements. Particles that enter at lower heights (Δ*y* = 0 ,.., 40 μm) experience a substantial vertical repulsive (upward) force prior to reaching the first element, which tends to extend their travel distance thereby increasing their capture time. Particles that enter at higher heights (Δ*y* = 70, …, 140 μm) bypass the first element, and therefore have extended trajectories and longer capture times.

The capture efficiency of this system can be estimated as follows. If we assume that the particles are uniformly distributed height-wise as they enter the microchannel, then the percentage of particles captured will be equal to the maximum predicted capture entry height 130 μm, divided by the total height of the microchannel, which is 200 μm. Based on this analysis, approximately 65% of the particles that enter the microsystem will be captured. The transport model Equations (25)–(27) can be used to optimize the capture efficiency of the system.

In addition to bioseparation as describe above, magnetic nanoparticles are also finding increasing use for cell sorting applications, especially at the microscale [34,39]. Immunomagnetic cell sorting involves the use of surface markers in the form of magnetic nanoparticles conjugated to antibodies, which are designed to bind to specific antibody receptors on target cells (magnetic labeling). The magnetically labeled cells are sorted using an applied field, which couples to the cellular-bound magnetic nanoparticles, thereby imparting a force to the cell enabling its controlled movement. To date, various continuous flow microfluidic cell sorters have been fabricated and characterized, and Newtonian particle transport models have been used to analyze their performance [39]. It should be noted that red blood cells have a sufficiently high magnetic susceptibility that they can be directly manipulated using a magnetic field, without the need for magnetic labeling [55]. Particle transport models have been successfully applied to immunomagnetic cell sorting, and magnetic manipulation of blood cells. Numerous systems have been analyzed using these methods including quadrupole cell sorters [34,55,56,57], continuous blood cell separators [58,59], and microfluidic-based blood cell separators [48,49,60].

### 4.3. Magnetic Drug Targeting

Conventional cancer treatments include surgery, chemotherapy, radiation, immunotherapy, and combinations thereof. The success of these therapies can be limited by various factors including inaccessibility of the tumor, surgical risk, deleterious side effects due to systemic distribution of toxic pharmaceuticals, and collateral irradiation of healthy tissue. An alternate and relatively new cancer treatment involves magnetic drug targeting wherein magnetic particles carrying anticancer agents are directed to malignant tissue using an applied field. This therapy can improve the effectiveness of the treatment while reducing undesired side effects. The growing application of magnetic targeting is due to rapid progress in the development of functionalized magnetic micro- and nanoparticles that are designed to target a specific tissue, and effect local chemo-, radio- and gene therapy at a tumor site [3,4,5,61,62,63,64].

**Figure 11:**
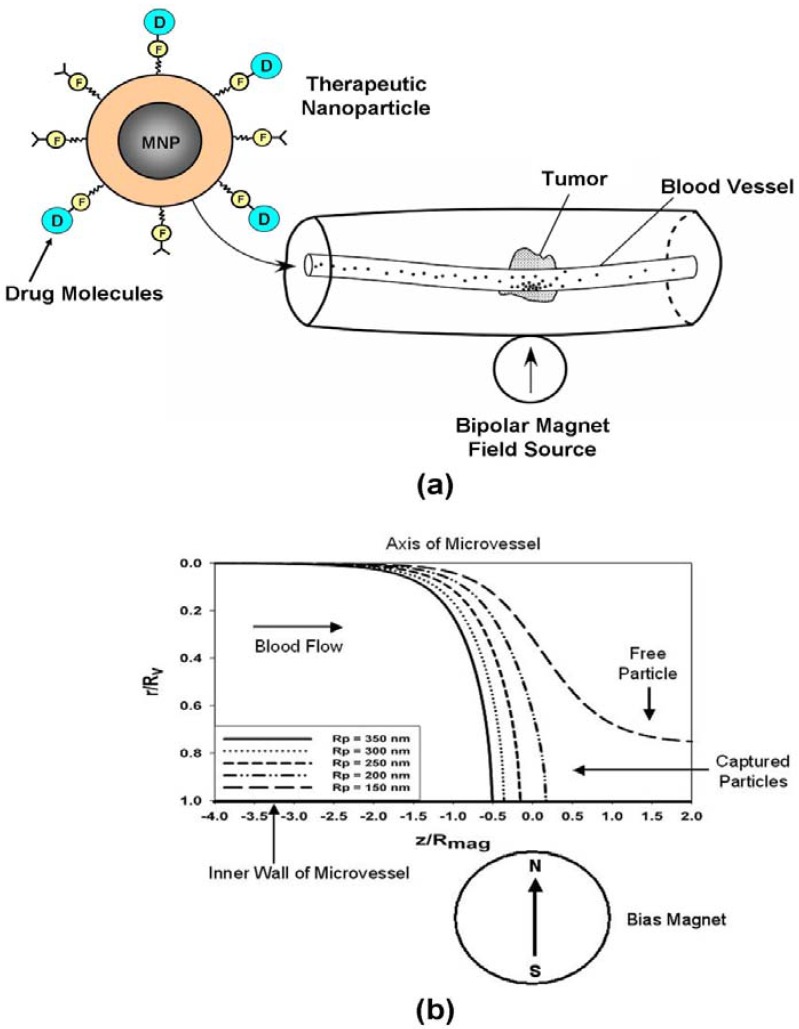
Magnetic drug targeting: (a) noninvasive drug targeting in a microvessel, (b) trajectories of Fe_3_O_4_ nanoparticles in a microvessel (cross-section of bias magnet shown for reference).

In magnetic drug targeting, the therapeutic agent can be either encapsulated into a magnetic micro or nanosphere or conjugated on its surface as shown in Figure 6 [5,65,66]. Magnetic particles with bound drug molecules are injected into the vascular system upstream from the malignant tissue (Figure 11a). They can be immobilized at the tumor site using a local magnetic field gradient produced by an external field source. Particle accumulation at the tumor is often augmented by magnetic agglomeration, and the efficiency of the accumulation depends on various physiological parameters including particle size, surface characteristics, field strength, blood flow rate, *etc*.

Drug targeting can be passive or active. In passive targeting, therapeutic particles “leak” out of the vascular system and accumulate at the tumor due to the enhanced permeability of the vasculature that is found near developing tumors. Specifically, most solid tumors exhibit a vascular pore cut-off size from 380–780 nm [5]. Tumor-limited lymphatic drainage adds to this process, and the combined effects result in selective accumulation of the particles in tumor tissue, which is known as enhanced permeation and retention.

In active drug targeting, magnetic particles are functionalized with molecules that are capable of selectively recognizing their target tissue and binding to it (Figure 6). Target recognition can occur at different levels, from whole organs/tissue to receptors on specific cells. Thus, therapeutic nanoparticles can provide treatment at both the organ/tissue and cellular level. In the latter case, they can be internalized and effect therapy within a cell *via* processes such as endocytoses or phagocytosis. Cellular targeting can be based on epitopes or receptors that are over- or exclusively expressed in tumor cells, or other over-expressed species such as low molecular weight ligands (folic acid, thiamine, sugars), proteins (transferrin, antibodies, lectins), peptides (RGD, LHRD), polysaccharides (hyaluronic acid), polyunsaturated fatty acids, and DNA, *etc*. The most common type of targeting molecules are antibodies, lectins, proteins, hormones, charged molecules and low molecular weight ligands such as folate [5,67].

Upon achieving a sufficient particle concentration at a tumor, drug molecules can be released from their carrier particles by changing physiological conditions such as pH, osmolality, or temperature, or by enzymatic activity [3,4]. Since the therapeutic agents are localized to regions of diseased tissue, higher dosages can be applied, which enables more effective treatment. This is in contrast to less selective conventional chemotherapy wherein a toxic drug is distributed systemically throughout the body, potentially harming healthy tissue.

Magnetic drug targeting has been studied using surface tumors [3,4,68], and small animal models including rabbits [68], swine [69,70], and rats [71,72]. The clinical trials have produced encouraging results that range from the permanent remission of squamous cell carcinoma in New Zealand White Rabbits [68], to the effective treatment of cancer in humans [73,74].

In addition to the clinical trials, theoretical research has been conducted to study the magnetic drug targeting process. Specifically, Newtonian and drift-diffusion transport models have been used to determine the viability of noninvasive therapy, and to design magnetic fields for clinical applications [26,31,75,76,77,78,79]. It is important to note that these studies are based on very simplistic models of blood flow (e.g., laminar fluid flow through a rigid blood vessel), and consequently they provide only a first-order estimate of the magnetic targeting process. In reality, blood vessels are complex flexible structures, and whole blood is a complex fluid consisting of a suspension of red and white blood cells, and platelets in plasma. Interesting computational research of blood flow based on medical images has been performed [80,81]. A rigorous analysis of particle transport through the vascular system needs to take these complexities into account and is beyond the scope of our discussion. Nevertheless, the transport models presented here enable estimates of particle trapping as a function of key variables including particle size, blood flow rate, location and strength of the field source, and the magnitude of the field gradient at the tumor site.

It is instructive to apply a transport model to a specific system. To this end, we consider a drug targeting system in which the particles are captured at the tumor site by a rare-earth cylindrical magnet positioned outside the body as shown in Figure 11a [26,31]. The magnet is vertically magnetized through its cross-section, and produces a nonuniform field distribution in nearby tissue. It is of infinite extent into the page (y direction), with a magnetization that is orthogonal to the blood flow. The magnetic force that it produces is based on an analytical expression for the field distribution within the microvessel, combined with a linear magnetization model for the magnetic response of particles as described by Equation (18). The fluidic analysis is based on the assumption of laminar blood flow through a cylindrical microvessel, and the effect of the blood cells is taken into account *via* use of an effective bulk viscosity *η* that depends on the hematocrit, which is the percentage by volume of packed red blood cells in a given sample of blood. The Newtonian transport equations for particle motion in the x-z plane the (z-axis is along the length of the microvessel) can be written in component form as

(29) $$m_{p}\frac{du_{px}}{dt}=-\mu_{0}V_{p}f(H_{a})M_{S}^{2}\;R_{mag}^{4}\;\frac{\left(x+d\right)}{2{\left({\left(x+d\right)}^{2}+z^{2}\right)}^{3}}-6\pi \eta R_{p}u_{px},$$

and

(30) $$m_{p}\frac{du_{pz}}{dt}=-\mu_{0}V_{p}f(H_{a})M_{S}^{2}\;R_{mag}^{4}\;\frac{z}{2{\left({\left(x+d\right)}^{2}+z^{2}\right)}^{3}}-6\pi \eta R_{p}\left[u_{pz}-2{\bar{u}}_{f}\left(1-\frac{x^{2}}{R_{v}^{2}}\right)\right]$$

where $R_{mag}$ is the radius of the magnet.

To demonstrate the theory, we choose a microvessel with a radius $R_{v}$ = 75 μm, and an average flow velocity ${\bar{u}}_{f}=15\;\text{mm}/\text{s}$. The magnet is 4 cm in diameter ($R_{mag}$ = 2.0 cm) with a magnetization $M_{s}=1\times 10^{6}\;\text{A}/\text{m}$. For the calculation of effective viscosity, we assume a hematocrit of 45% [26]. The initial conditions for the particles are as follows. They start on the axis of the microvessel, far enough upstream so that the magnetic force is initially negligible. They have an initial velocity equal to the axial flow velocity (*i.e.*, $u_{x}(0)=0$, $u_{y}(0)=0$, and $u_{z}(0)=2{\bar{u}}_{f}=30\;\text{mm}/\text{s}$). We predict the trajectories of five different sized Fe_3_O_4_ particles: $R_{p}$ = 150, 200, 250, 300 and 350 nm, for a magnet-to-vessel distance d = 2.5 cm (*i.e.* the center of the microvessel is 0.5 cm from the surface of the magnetic). In these plots the radial position r=|x| of the particle is normalized with respect to the microvessel radius $R_{v}$, and the axial position z is normalized with respect to the magnet radius $R_{mag}$. The predicted trajectories along with the magnetic geometry, are shown in Figure 11b. This plot shows that all the particles except the smallest ($R_{p}$ = 150 nm) are captured by the magnet. The predictions take only a few minutes to complete, and the model can be used to estimate capture efficiency as a function of key variables including the particle size and magnetic properties, and the flow rate *etc*. To date, various studies have shown that noninvasive magnetic targeting can be achieved using submicron Fe_3_O_4_ particles, when the target tissue is within several centimeters from the surface of the body.

### 4.4. Magnetofection

Magnetofection is a relatively new process, and interest in it is growing rapidly. In magnetofection, magnetic carrier particles with surface-bound gene vectors are magnetically attracted towards target cells for transfection or transduction. Transfection broadly refers to the process of introducing nucleic acids into cells by non-viral methods, respectively [36]. It should be noted that magnetofection also works well for transduction, wherein nucleic acids are introduced into cells using viral vectors.

**Figure 12:**
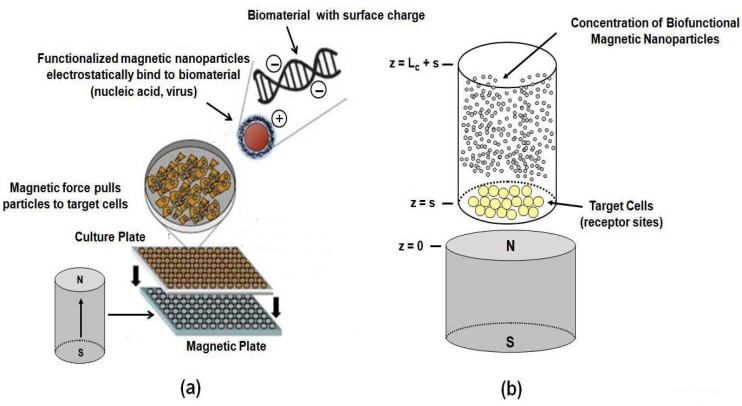
Magnetofection: (a) culture plate with an array of cell cultures positioned above a magnetic plate with an array of cylindrical rare-earth magnets, (b) a single cell culture with nanoparticles with surface-bound gene vectors (adapted with permission from reference [24]).

In a typical *in vitro* magnetofection system the target cells are located at the bottom of a fluidic chamber and a rare-earth magnet beneath the chamber provides a magnetic force that attracts the biofunctional particles towards the cells as shown in Figure 12 [36,61,82]. Magnetofection has distinct advantages over traditional transfection methods. Specifically, high transfection rates are obtained with significantly low vector doses, and the process time is dramatically reduced, from hours to minutes [83,84]. However, despite the advantages and growing use of magnetofection, relatively few authors have studied particle transport and accumulation for this process.

We apply the drift-diffusion transport model to analyze the performance of a conventional magnetofection system [24]. We study the accumulation of Fe_3_O_4_ nanoparticles at the base of a cylindrical fluidic chamber positioned above a rare-earth NdFeB magnet. We assume that the chamber has a radius $R_{c}$ = 2 mm and length $L_{c}$ = 3 mm, and that it is positioned 1 mm above the magnet. The magnet has a radius $R_{m}$ = 2.5 mm and length $L_{m}$ = 5 mm and is magnetized to saturation, $M_{s}=8\times 10^{5}\;\text{A}/\text{m}$ ($B_{r}$ = 1T). The chamber and magnet dimensions are representative of a 96 well plate magnetofection system. We further assume that the fluid in the chamber is nonmagnetic ($\chi_{f}=0$) with a viscosity and density equal to that of water, η=0.001 N∙s/m^2^, and $\rho_{f}=1000$ kg/m^3^∙ Fe_3_O_4_ nanoparticles have a density $\rho_{p}=5000$ kg/m^3^ and a saturation magnetization $M_{sp}=4.78\times 10^{5}\;\text{A}/\text{m}$. We also assume that the hydrodynamic radius of a particle is the same as its physical radius, $R_{p}=R_{p,hyd}$. We adopt a magnetization model as in Equation (28).

**Figure 13:**
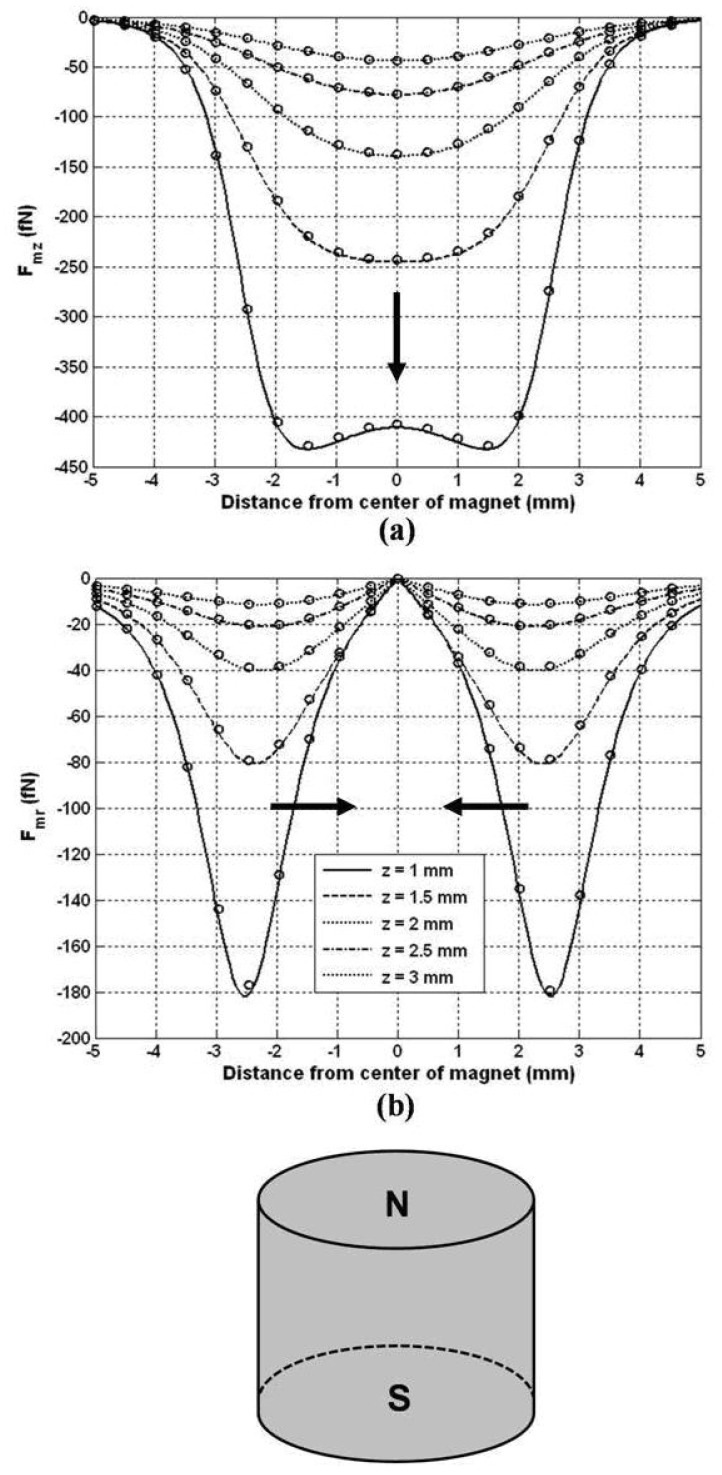
Magnetic force above a cylindrical rare-earth magnet (arrows show direction of force, ο = FEA): (a) F_mz_, and (b) F_mr_.

First, we compute the magnetic force along a series of horizontal lines $0\leq r\leq 2R_{m}$ corresponding to different heights z = 1, 1.5, 2, 2.5 and 3 mm above the magnet. We choose a particle radius $R_{p}$ = 100 nm and evaluate the force using closed-form expressions [24]. The predicted force values are compared with corresponding numerical data obtained using finite element analysis (FEA) in Figure 13.

The magnitude of the force varies across the chamber and decreases with distance from the magnet. The maximum force on the particle is sub nano-Newton, but substantially greater forces can be achieved using larger particles as the force scales with $R_{p}^{3}$. The axial force $F_{mz}$ attracts particles downward towards the surface of the magnet as indicated by the force arrow in Figure 13a. Farther from the magnet $F_{mz}$ is strongest along the z-axis (centerline of the magnet). However, closer to the magnet it peaks off-axis, which implies that the particles will have a pronounced accumulation in an annulus at the bottom of chamber. The radial force $F_{mr}$ peaks (in a negative sense) above the radial edge of the magnet and acts to move the particles radially inward towards the z-axis, as indicated by the force arrows in Figure 13b. Thus, particle accumulation is expected to be focused toward the center of the chamber and minimal near the edge of the magnet. It should be noted that this analysis ignores the effects (*i.e.,* field and force) of the neighboring magnets in an array plate as shown in Figure 12a. These effects can be neglected when the spacing between the magnets is large relative to the distance from a magnet to its respective cell culture.

Next, we perform two 1D parametric studies to understand particle accumulation as a function of particle size, and magnet-to-chamber spacing, respectively. In the 1D approximation, the field and force are strictly in the z-direction, *i.e.,* no radial components. This approximation is reasonable when the diameter of the magnet is much greater than the diameter and height of the fluidic chamber. The 1D (on-axis) field solution for a cylindrical rare-earth magnet that is magnetized to a saturation level $M_{s}$ is well-known (p 129 in [30]),

(31) $$H_{az}(z)=\frac{M_{s}}{2}\left[\frac{z+L_{m}}{\sqrt{{\left(z+L_{m}\right)}^{2}+R_{m}^{2}}}-\frac{z}{\sqrt{z^{2}+R_{m}^{2}}}\right]$$

where z is the distance above the top of the magnet, and $L_{m}$ and $R_{m}$ are the length and radius of the magnet, respectively. The magnetic force along the z-axis above the magnet is given by [24]

(32) $$F_{mz}(z)=\mu_{0}V_{p}\;f(H_{a})\frac{M_{s}^{2}R_{m}^{2}}{4}\left[\frac{\left(z+L_{m}\right)}{{\left({\left(z+L_{m}\right)}^{2}+R_{m}^{2}\right)}^{2}}+\frac{z}{{\left(z^{2}+R_{m}^{2}\right)}^{2}}-\frac{\left(z+L_{m}\right)\left({\left(z+L_{m}\right)}^{2}+R_{m}^{2}\right)+z\left(z^{2}+R_{m}^{2}\right)}{{\left(z^{2}+R_{m}^{2}\right)}^{3/2}{\left({\left(z+L_{m}\right)}^{2}+R_{m}^{2}\right)}^{3/2}}\right]$$

For the 1D analysis we solve the drift-diffusion equation

(33) $$\frac{\partial c}{\partial t}=\frac{\partial }{\partial z}(D\frac{\partial c}{\partial z}-Uc)$$

in the fluid chamber using the numerical finite volume method (FVM). The computational domain spans the length of the chamber $z_{c,b}\leq z\leq z_{c,t}$, where $z_{c,t}$ and $z_{c,b}$ denote the top and bottom of the chamber, respectively. The drift velocity is a function of the position in the chamber, $U(z)=\gamma F_{m}(z)$. The FVM discretization of Equation (33) at an interior node is

(34) $$c_{i}^{n+1}=c_{i}^{n}-\frac{\delta t}{\delta z}(P_{i+1/2}-P_{i-1/2})\;(i=1,2,...,N_{z})$$

where $\delta z=L_{c}/N_{z}$ is the length of a computational cell and $L_{c}$ is the length of the chamber. $c_{i}^{n}$ and $c_{i}^{n+1}$ are the values of the concentration at the i’th computational node at time steps n and n+1, respectively. $P_{i\pm 1/2}$ is a discretized version of the particle flux $\;-\left(D\frac{\partial c}{\partial z}-Uc\;\right)$ at the edges of the computational cell $z_{i\pm 1/2}$. We use an upwind scheme and obtain

(35) $$P_{i+1/2}=-\left[D\frac{c_{i+1}^{n}-c_{i}^{n}}{\delta z}-(\min(U_{i},0)c_{i}^{n}+\max(U_{i-1},0)c_{i-1}^{n})\right]$$

The magnetic field solution Equation **(31)** applies when the top of the magnet is at z=0, and therefore $z_{c,t}=L_{c}+s$ and $z_{c,b}=s$ as shown in Figure 12b. We solve Equation (34) subject to an initial condition in which there is a uniform particle volume concentration throughout the chamber $c(z,0)=c_{0}$. We apply a zero-flux Neumann boundary condition at the top of the chamber and a Dirichlet condition $c(z_{c,b},t)=0$ at the bottom. The latter condition mimics the magnetofection process wherein nanoparticles that reach the bottom of the chamber are removed from the computation as it is assumed that they bind with receptor sites on target cells and therefore no longer influence particle transport. It is assumed that there are a sufficient number of receptors to accommodate all of the particles in the chamber. We compute particle accumulation by summing the number of particles that reach the base of the chamber during each time step.

**Figure 14:**
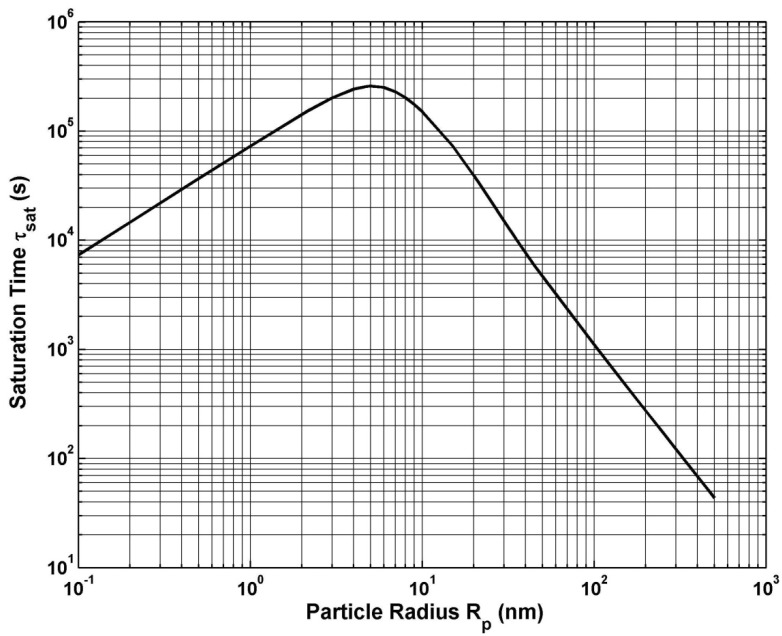
Particle saturation time τ_sat_ *versus* particle radius R_p_.

In the first 1D study we compute particle accumulation as a function of particle radius for $R_{p}$ = 0.1–500 nm. For each radius we determine the time required for all of the particles to reach the bottom of the chamber; we call this the saturation time $\tau_{sat}$. As shown in Figure 14, our analysis indicates that there are two distinct modes of transport: diffusion-dominated transport, which applies for smaller particles ($R_{p}$ <5 nm); and drift-dominated transport, which applies for larger particles (*R_p_* > 5 nm). We find that $\tau_{sat}$ increases with $R_{p}$ in diffusion-dominated transport, but decreases with $\frac{1}{R_{p}^{2}}$ in drift-dominated transport. The first result can be understood by considering the time $\tau_{dif}$ required to a particle to diffuse a distance d, $\tau_{dif}\propto d^{2}/D$; it follows that $\tau_{dif}\propto \;R_{p}$. This is in contrast to drift-dominated transport wherein larger particles have a higher drift velocity. Specifically, to first order $u_{p}=\gamma F_{m}$ and from Equation (32), and the definition of the mobility, it follows that $v_{p}\propto R_{p}^{2}$, which implies that $\tau_{sat}\propto \frac{1}{R_{p}^{2}}$. Practical magnetofection systems operate in a drift-dominated mode with particle sizes that range from 100 to 1000 nm in diameter (see www.bocascientific.com). The accumulation rates for these systems are orders of magnitude faster than diffusion-limited accumulation [83,84].

**Figure 15:**
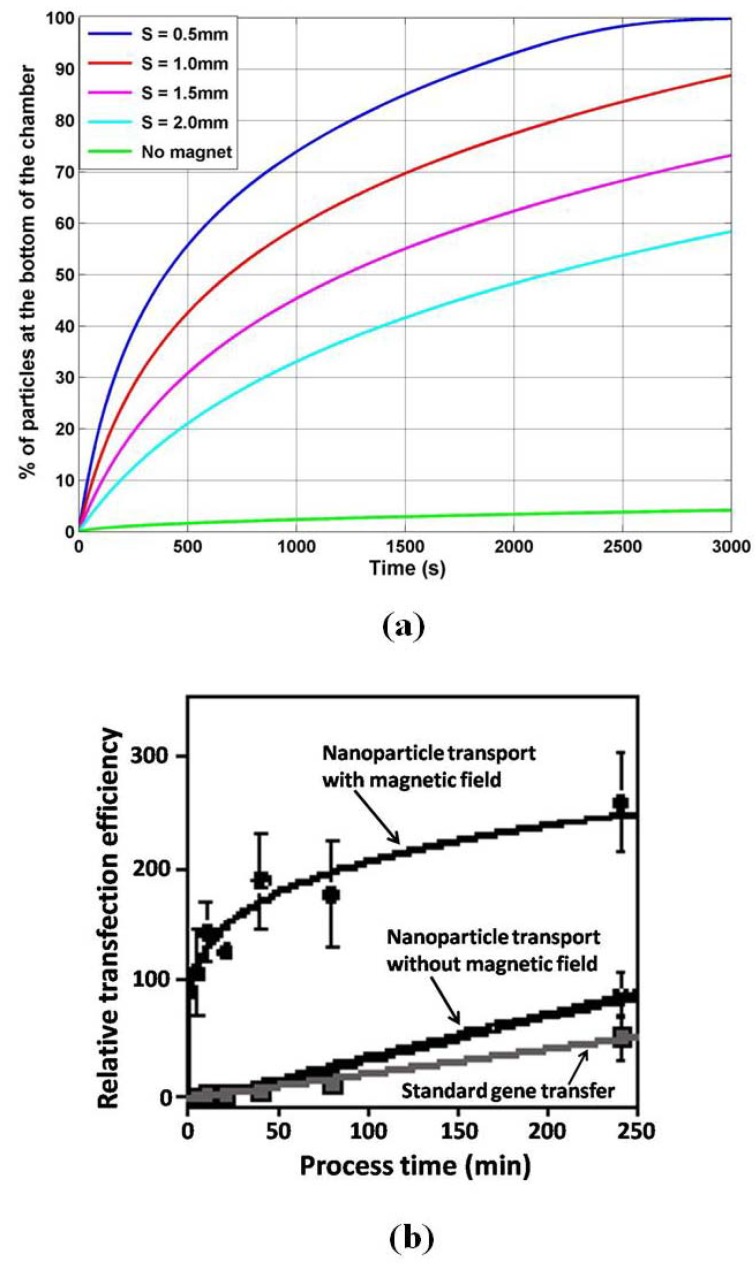
Nanoparticle accumulation: (a) percent of particles at the base of the chamber *versus* time as a function of magnet-to-chamber spacing s, (b) measured relative transfection efficiency, with and without an applied field (adapted with permission from reference [84]).

In the second 1D study we fix the particle radius $R_{p}$ = 50 nm and compute particle accumulation as a function of the separation s between the magnet and the chamber. From our previous analysis we know that the transport of this particle is drift-dominated. We study particle accumulation for s = 0.5, 1.0, 1.5, and 2.0 mm, and we compare this with diffusion-limited accumulation, *i.e.,* with no applied field (Figure 15a). We find that the rate of magnetically induced accumulation is orders of magnitude faster than that of diffusion-limited (no field) accumulation, which is consistent with experimental data shown in Figure 15b. Furthermore, the rate of accumulation increases substantially with decreasing separation. The transport model enables rapid parametric analysis of particle accumulation, which is useful for optimizing novel magnetofection systems.

## 5. Conclusions

We have provided an overview of biofunctional magnetic nanoparticles and their use as transport agents in bioapplications. We have summarized the basic properties of these particles and their functionalization for bioapplications. We have also discussed the physics governing particle motion in a viscous medium under the influence of an applied field (magnetophoresis). We have presented two different transport models that describe the magnetophoretic behavior of magnetic particles at two different scales: a Newtonian transport model for larger (submicron-micron) particles, and a drift-diffusion transport model for smaller (nanoscale) particles that takes into account Brownian motion. We have discussed specific bioapplications including bioseparation, magnetic drug targeting and magnetofection. We have demonstrated the use of the transport models *via* application to these processes.

As for the future, the use of magnetic particles in bioapplications is in its infancy, and will undoubtedly grow significantly in the coming years. Methods for synthesizing, functionalizing and sensing these particles are advanced rapidly, giving rise to a proliferation of new applications. The small size, large surface to volume ratio, inherent multifunctional nature (magnetic manipulation and RF absorption) and unique superparamagnetic behavior of magnetic nanoparticles makes them well suited for bioapplications, both *in vitro* and *in vivo* [85,86].

The most significant advances in future applications of magnetic particles will likely involve the use of microfluidic systems in which magnetic particles will transport or immobilize a biomaterial to facilitate its detection or analysis. The advantages of microfluidic-based analytical systems are numerous [45,86]. Specifically, they function with very small sample sizes, and are capable of rapid processing and a high level of automation with high reliability and low cost. Currently, much research is devoted to the development of multifunctional “Micro-Total Analysis Systems” (µTAS) that are capable of preprocessing and separating biosamples, mixing them with desired reagents, and selectively subjecting them to specific testing and detection. Near term, such devices will find increasing use in clinical point-of-service applications for medical diagnosis and disease monitoring. In the long term, they will advance fundamental understanding in fields such as genomics and proteomics by enabling massively parallel biochemical processing of biomaterials down to the single molecule level. The realization of practical lab-on-a-chip systems for biomedical applications will revolutionize medical diagnostics and treatment with far reaching benefits to society as a whole.

## References

[1] Pankhurst Q.A., Thanh N.K.T., Jones S.K., Dobson J. (2009). Progress in applications of magnetic nanoparticles in biomedicine. J. Phys. D: Appl. Phys..

[2] Pankhurst Q.A., Connolly J., Jones S.K., Dobson J. (2003). Applications of magnetic nanoparticles in biomedicine. J. Phys. D: Appl. Phys..

[3] Berry C.C. (2009). Progress in functionalization of magnetic nanoparticles for applications in biomedicine. J. Phys. D: Appl. Phys..

[4] Berry C.C., Curtis A.S.G. (2003). Functionalization of magnetic nanoparticles for applications in biomedicine. J. Phys. D: Appl. Phys..

[5] Arrueboa M., Fernández-Pachecoa R., Ibarraa R.M., Santamaría J. (2007). Magnetic nanoparticles for drug delivery. Nanotoday.

[6] Majewski P., Thierry B. (2007). Functionalized magnetite nanoparticles - synthesis, properties, and bio-Applications. Crit. Rev. Solid State Mater. Sci..

[7] Pedro T., Morales M.P., Veintemillas-Verdaguer S., Gonzalez-Carreno T., Serna C.J. (2003). The preparation of magnetic nanoparticles for applications in biomedicine. J. Phys. D: Appl. Phys..

[8] Safarik I., Safarikova M. (2002). Magnetic nanoparticles and biosciences. Monatshefte fur Chem..

[9] Moroz P., Jones S.K., Gray B.N. (2002). Magnetically mediated hyperthermia: current status and future directions. Int. J. Hyperthermia.

[10] Hergt R., Duzt S. (2007). Magnetic particle hyperthermia-biophysical limitations of a visionary tumor therapy. J. Magn. Magn. Mater..

[11] Gupta A.K., Gupta M. (2005). Synthesis and surface engineering of iron oxide nanoparticles for biomedical applications. Biomaterials.

[12] Kircher M.F., Mahmood U., King R.S., Weissleder R., Josephson L. (2003). A Multimodal Nanoparticle for Preoperative Magnetic Resonance Imaging and Intraoperative Optical Brain Tumor Delineation. Cancer Res..

[13] Sahoo Y., Goodarzi A., Swihart M.T., Ohulchanskyy T.Y., Kaur N., Furlani E.P., Prasad P.N. (2005). Ferrofluid of magnetite nanoparticles: fluorescence labeling and magnetophoretic control. J. Phys. Chem. B..

[14] Sosnovik D.E., Nahrendorf M., Weissleder R. (2008). Magnetic nanoparticles for MR imaging: agents, techniques and cardiovascular applications. Basic Res. Cardiol..

[15] Prasad P. N. (2005). Introduction to Biophotonics.

[16] Levy L., Sahoo Y., Kim K.-S., Bergey E.J., Prasad P.N. (2002). Nanochemistry: synthesis and characterization of multifunctional nanoclinics for biological applications. Chem. Mater..

[17] Medarova Z., Pham W., Kim Y., Dai G., Moore A. (2006). *In vivo* imaging of tumor response to therapy using a dual-modality imaging strategy. Int. J. Cancer..

[18] Lu A-H., Salabas E.L., Schüth F. (2007). Magnetic nanoparticles: synthesis, protection, functionalization, and application. Angew. Chem. Int. Ed..

[19] Willard M.A., Kurihara L.K., Carpenter E.E., Calvin S., Harris V.G. (2004). Chemically prepared magnetic nanoparticles. Int. Mats. Rev..

[20] Sahoo Y., Cheon M., Wang S., Luo H., Furlani E.P., Prasad P.N. (2004). Field-directed self-assembly of magnetic nanoparticles. J. Phys. Chem. B.

[21] Sahoo Y., He Y., Swihart M.T., Wang S., Luo H., Furlani E.P., Prasad P.N. (2005). An aerosol mediated magnetic colloid: study of nickel nanoparticles. J. Appl. Phys..

[22] Furlani E.P. (2006). Analysis of particle transport in a magnetophoretic microsystem. J. Appl. Phys..

[23] Gerber R., Takayasu M., Friedlander F.J. (1983). Generalization of HGMS theory: the capture of ultrafine particles. IEEE Trans. Magn..

[24] Furlani E.P., Ng K.C. (2008). Nanoscale magnetic biotransport with application to magnetofection. Phys. Rev. E.

[25] Fletcher D. (1991). Fine particle high gradient magnetic entrapment. IEEE Trans. Magn..

[26] Furlani E.P., Ng K.C. (2006). Analytical model of magnetic nanoparticle capture in the microvasculature. Phys. Rev. E.

[27] Sinha A., Ganguly R., De A.K., Puri I.K. (2007). Single magnetic particle dynamics in a microchannel. Phys. Fluids.

[28] Furlani E.P., Sahoo Y. (2006). Analytical model for the magnetic field and force in a magnetophoretic microsystem. J. Phys. D: Appl. Phys..

[29] Furlani E.P., Sahoo Y., Ng K.C., Wortman J.C., Monk T.E. (2007). A model for predicting magnetic particle capture in a microfluidic bioseparator. Biomed. Microdev..

[30] Furlani E. P. (2001). Permanent Magnet and Electromechanical Devices; Materials, Analysis and Applications.

[31] Furlani E.J., Furlani E.P. (2007). A model for predicting magnetic targeting of multifunctional particles in the microvasculature. J. Magn. Magn. Mat..

[32] Liu C., Lagae L., Wirix-Speetjens R., Borghs G. (2007). On-chip separation of magnetic particles with different magnetophoretic mobilities. J. Appl. Phys..

[33] Williams P.S., Carpino F., Zborowski M. (2009). Magnetic nanoparticle drug carriers and their study by quadrupole magnetic field-flow fractionation. Mol. Pharmaceutics.

[34] Zborowski M., Chalmers J.J. (2007). Magnetic Cell Separation.

[35] Mykhaylyk O., Antequera Y.S., Vlaskou D., Plank C. (2007). Generation of magnetic nonviral gene transfer agents and magnetofection *in vitro*. Nat. Protocols.

[36] Schillinger U., Brilla T., Rudolph C., Huth S., Gersting S., Krotz F., Hirschberger J., Bergemann C., Plank C. (2005). Review - advances in magnetofection-magnetically guided nucleic acid delivery. J. Magn. Magn. Mat..

[37] Stamenovic D., Wang N. (2000). Cellular response to mechanical stress: invited review: engineering approaches to cytoskeletal mechanics. J. Appl. Physiol..

[38] Safarik I., Safarikova M. (2004). Magnetic techniques for the isolation and purification of proteins and peptides. BioMagn. Res. Technol..

[39] Pamme N., Wilhelmb C. (2006). Continuous sorting of magnetic cells *via* on-chip free-flow magnetophoresis. Lab Chip.

[40] Molday R.S., Yen S.P.S., Rembaum A. (1997). Application of magnetic microspheres in labeling and separation of cells. Nature.

[41] Nam J-M., Thaxton C.S., Mirkin C.A. (2003). Nanoparticle-based bio–bar codes for the ultrasensitive detection of proteins. Science.

[42] Lee H., Sun E., Ham D., Weissleder R. (2008). Chip-NMR biosensor for detection and molecular analysis of cells. Nat. Med..

[43] Yazdankhah S.P., Slverd L., Simonsen S., Olsen E. (1999). Development and evaluation of an immunomagnetic separation-ELISA for the detection of Staphylococcus aureus thermostable nuclease in composite milk. Vet. Microbiol..

[44] Safarýk I., Safarýkova M., Hafeli U., Schutt W., Teller J., Zborowski M. (1997). Scientific and Clinical Applications of Magnetic Carriers.

[45] Hardt S., Friedhelm S. (2007). Microfluidic Technologies for Miniaturized Analysis Systems.

[46] Pamme N. (2006). Magnetism and microfluidics. Lab Chip.

[47] Gijs M.A.M. (2004). Magnetic bead handling on-chip: new opportunities for analytical applications. Microfluid Nanofluid..

[48] Han K.H., Frazier A.B. (2005). Diamagnetic capture mode magnetophoretic microseparator for blood cells. J. Micromech. Sys..

[49] Han K.H., Frazier A.B. (2004). Continuous magnetophoretic separation of blood cells in microdevice format. J. Appl. Phys..

[50] Choi J-W., Liakopoulos T.M., Ahn C.H. (2001). An On-Chip Magnetic Bead Separator Using Spiral Electromagnets with Semi-Encapsulated Permalloy. Biosens. Bioelectron..

[51] Choi J-W., Ahn C.H., Bhansali S., Henderson H.T. (2000). A new magnetic bead-based, filterless bio-separator with planar electromagnet surfaces for integrated bio-detection systems. Sens. Actuator..

[52] Furlani E.P., Kumar C. (2010). Particle transport in magnetophoretic microsystems. Microfluidic Devices in Nanotechnology: Current Status and Future Perspective.

[53] Bu M., Christensen T.B., Smistrup K., Wolff A., Hansen M.F. (2008). Characterization of a microfluidic magnetic bead separator for high-throughput applications. Sens. Actuator. A.

[54] Gijs M.A.M. (2010). Microfluidic applications of magnetic particles for biological analysis and catalysis. Chem. Rev..

[55] Zborowski M., Ostera G.R., Moore L.R., Milliron S., Chalmers J.J., Schechter A.N. (2003). Red blood cell magnetophoresis. Biophys. J..

[56] Zborowski M., Sun L., Moore L.R., Williams S., Chalmers J.J. (1999). Continuous cell separation using novel magnetic quadrupole flow sorter. J. Magn. Magn. Mater..

[57] Sun L., Zborowski M., Moore L.R., Chalmers J.J. (1998). Continuous, flow-through immunomagnetic cell sorting in a quadrupole field. Cytometry A.

[58] Takayasu M., Kelland D.R., Minervini J.V. (2000). Continuous magnetic separation of blood components from whole blood. IEEE Trans. Appl. Supercond..

[59] Takayasu M., Duske N., Ash S.R., Friedlaender F. (1982). HGMS studies of blood–cell behavior in plasma. IEEE Trans. Magn..

[60] Furlani E.P. (2007). Magnetophoretic separation of blood cells at the microscale. J. Phys. D: Appl. Phys..

[61] Dobson J. (2006). Magnetic micro- and nano-particle-based targeting for drug and gene delivery. Nanomedicine.

[62] Ferrari M. (2005). Cancer nanotechnology: opportunities and challenges. Nat. Rev. Cancer.

[63] Marcucci F., Lefoulon F. (2004). Active targeting with particulate drug carriers in tumor therapy: fundamentals and recent progress. Drug Disc. Today.

[64] Davis S.S. (1997). Biomedical applications of nanotechnology-implications for drug targeting and gene therapy. Trends Biotechnol..

[65] Zheng Q., Ohulchanskyy T.Y., Sahoo Y., Prasad P.N. (2007). Water-dispersible polymeric structure co-encapsulating a novel hexa-peri-hexabenzocoronene core containing chromophore with enhanced two-photon absorption and magnetic nanoparticles for magnetically guided two-photon cellular imaging. J. Phys. Chem. C.

[66] Cinteza L.O., Ohulchanskyy T.Y., Sahoo Y., Bergey E.J., Pandey R.K., Prasad P.N. (2006). Diacyllipid micelle-based nanocarrier for magnetically guided delivery of drugs in photodynamic therapy. Mol. Pharm..

[67] Sudimack J., Lee R.J. (2000). Targeted drug delivery *via* the folate receptor. Adv. Drug Deliv. Rev..

[68] Alexiou C., Arnold W., Klein R.J., Parak F. G., Hulin P., Bergemann C., Erhardt W., Wagenpfeil S., Lübbe A.S. (2000). Locoregional cancer treatment with magnetic drug targeting. Cancer Res..

[69] Goodwin S.C., Bittner C.A., Peterson C.L., Wong G. (2001). Single-dose toxicity study of hepatic intra-arterial infusion of doxorubicin coupled to a novel magnetically targeted drug carrier. Toxicol. Sci..

[70] Goodwin S.C., Peterson C., Hoh C., Bittner C. (1999). Targeting and retention of magnetic targeted carriers (MTCs) enhancing intra-arterial chemotherapy. J. Magn. Magn. Mater..

[71] Pulfer S.K., Gallo J.M. (1999). Enhanced brain tumor selectivity of cationic magnetic polysaccharide microspheres. J. Drug Target..

[72] Pulfer S.K., Ciccotto S.L., Gallo J.M. (1999). Distribution of small magnetic particles in brain tumor-bearing rats. J. Neuro-Oncol..

[73] Lubbe A.S., Alexiou C., Bergemann C. (2001). Clinical applications of magnetic drug targeting. J. Surg. Res..

[74] Lubbe A.S., Bergemann C., Riess H., Schriever F., Reichardt P., Possinger K., Matthias M., Dörken B., Herrmann F., Gürtler R., Hohenberger P., Haas N., Sohr R., Sander B., Lemke A.-J., Ohlendorf D., Huhnt W., Huhn D. (1996). Clinical experiences with magnetic drug targeting: a phase I study with 4'-epidoxorubicin in 14 patients with advanced solid tumors. Cancer Res..

[75] Aviles M.O., Ebner A.D., Chen H.T., Rosengart A.J., Kaminskic M.D., Ritter J. A. (2005). Theoretical analysis of a transdermal ferromagnetic implant for retention of magnetic drug carrier particles. J. Magn. Magn. Mat..

[76] Yellen B.B., Forbesb Z.G., Halversona D.S., Fridmanb G., Barbeea K.A., Chornyc M., Levyc R., Friedman G. (2005). Targeted drug delivery to magnetic implants. J. Magn. Magn. Mat..

[77] Chen H.T., Ebner A.D., Rosengart A.J., Kaminski M.D., Ritter J. (2004). Analysis of magnetic drug carrier particle capture by a magnetizable intravascular stent: 1: parametric study with single wire correlation. J. Magn. Magn. Mat..

[78] Chen H.T., Ebner A.D., Kaminski M.D., Rosengart A.J., Ritter J. (2005). Analysis of magnetic drug carrier particle capture by a magnetizable intravascular stent-2: parametric study with multi-wire two-dimensional model. J. Magn. Magn. Mat..

[79] Ritter J.A., Ebner A.D., Daniel K.D., Stewart K.L. (2004). Application of high gradient magnetic separation principles to magnetic drug targeting. J. Magn. Magn. Mat..

[80] Linninger A.A., Somayaji M.R., Mekarski M., Zhang L. (2008). Prediction of convection enhanced drug delivery to the human brain. J. Theor. Biol..

[81] Linninger A.A., Xenos M., Sweetman B., Ponkshe S., Guo X., Penn R. (2009). A mathematical model of blood, cerebrospinal fluid and brain dynamics. J. Math. Bio..

[82] Dobson J. (2006). Gene therapy progress and prospects: magnetic nanoparticle-based gene delivery. Gene Ther..

[83] Plank C., Schillinger U., Scherer F., Bergemann C., Rémy J.S., Krötz F., Anton M., Lausier J., Rosenecker J. (2003). The magnetofection method: using magnetic force to enhance gene therapy. Biol. Chem..

[84] Plank C., Scherer F., Schillinger U., Anton M., Bergemann C. (2002). Magnetofection: enhancing and targeting gene delivery by magnetic force. Eur. Cells Mat..

[85] Prasad P. N. (2004). Nanophotonics.

[86] Nguyen N-T., Wereley S.T. (2006). Fundamentals and Applications of Microfluidics.

